# Profiles of miRNAs matched to biology in aromatase inhibitor resistant breast cancer

**DOI:** 10.18632/oncotarget.12103

**Published:** 2016-09-17

**Authors:** Reiner Hoppe, Ping Fan, Florian Büttner, Stefan Winter, Amit K. Tyagi, Heather Cunliffe, V. Craig Jordan, Hiltrud Brauch

**Affiliations:** ^1^ Dr. Margarete Fischer-Bosch-Institute of Clinical Pharmacology, Stuttgart, Germany; ^2^ University of Tübingen, Tübingen, Germany; ^3^ Department of Breast Medical Oncology, MD Anderson Cancer Center, University of Texas, Houston, TX, USA; ^4^ German Cancer Consortium (DKTK) and German Cancer Research Center (DKFZ), Heidelberg, Germany; ^5^ Department of Pathology, Dunedin School of Medicine, University of Otago, Dunedin, New Zealand

**Keywords:** breast cancer, AI resistance, microRNA profiling, pathway enrichment, E_2_-inducible apoptosis

## Abstract

Aromatase inhibitor (AI) resistance during breast cancer treatment is mimicked by MCF-7:5C (5C) and MCF-7:2A (2A) cell lines that grow spontaneously. Survival signaling is reconfigured but cells are vulnerable to estradiol (E_2_)-inducible apoptosis. These model systems have alterations of stress related pathways including the accumulation of endoplasmic reticulum, oxidative, and inflammatory stress that occur prior to E_2_-induced apoptosis. We investigated miRNA expression profiles of 5C and 2A to characterize their AI resistance phenotypes. Affymetrix GeneChip miRNA2.0 arrays identified 184 miRNAs differentially expressed in 2A and 5C compared to E_2_-free wild-type MCF-7:WS8. In 5C, 34 miRNAs of the DLK1-DIO3 locus and miR-31 were overexpressed, whereas miR-222 was low. TCGA data revealed poor and favorable overall survival for low miR-31 and miR-222 levels, respectively (HR=3.0, 95% CI:1.9-4.8; HR=0.3, 95% CI:0.1-0.6). Targets of deregulated miRNAs were identified using CLIP-confirmed TargetScan predictions. KEGG enrichment analyses for 5C- and 2A-specific target gene sets revealed pathways associated with cell proliferation including insulin, mTOR, and ErbB signaling as well as immune response and metabolism. Key genes overrepresented in 5C- and 2A-specific pathway interaction networks including *EGFR*, *IGF1R* and *PIK3R1* had lower protein levels in 5C compared to 2A and were found to be differentially modulated by respective miRNA sets. Distinct up-regulated miRNAs from the DLK1-DIO3 locus may cause these attenuative effects as they are predicted to interact with corresponding 3′ untranslated regions. These new miRNA profiles become an important regulatory database to explore E_2_-induced apoptotic mechanisms of clinical relevance for the treatment of resistant breast cancer.

## INTRODUCTION

Long-term estrogen deprivation is the standard of care in patients with estrogen receptor (ER)-positive breast cancer. Two proven treatment options exist: tamoxifen, a selective ER modulator which blocks 17ß-estradiol (E_2_) binding to ER to stop tumor growth, and aromatase inhibitors (AI), which block the aromatase enzyme that prevents the conversion of androgens to estrogens. Despite their well-established effectiveness [1, 2], patients frequently display *de novo* or acquired resistance which ultimately leads to disease progression and death.

Laboratory models of long-term E_2_-deprived breast cancer cells provide a valuable surrogate approach to study clinical AI resistance. The goal is to discover biomarkers and new therapeutic targets to subvert AI resistance. Long-term E_2_-deprivation selects for tumor cells that can grow spontaneously, but eventually, through clonal selection, become vulnerable to E_2_-inducible apoptosis [3]. Knowledge of the mechanisms of endocrine resistance originally evolved from *in vivo* studies in which MCF-7 cells were inoculated into athymic mice treated with E_2_ for tumor growth and tamoxifen for growth inhibition. Endocrine resistance evolves over time and manifests in phase I and phase II of which the latter refers to the cellular reprogramming towards E_2_-inducible apoptosis, for which clinical evidence exists.

Phase I-resistance occurs after one year of tamoxifen treatment *in vivo* in that growth becomes dependent on tamoxifen or estradiol [4, 5]. Clinical correlates are disease progression during tamoxifen therapy with breast cancer recurrence and development of metastasis. Phase II-resistance develops during 5 years of continuous passage of MCF-7 tumors in tamoxifen-treated athymic mice and refers to the reconfiguration of survival signaling [5]. While tumors still grow in response to tamoxifen treatment, they now rapidly regress with E_2_, referred to as E_2_-induced apoptosis. Clinical correlates are the successful treatment of metastatic and endocrine refractory breast cancer with high and/or low dose E_2_. Although estrogen was the first chemotherapy applied to cancer more than 70 years ago, it is recently being re-investigated with clinical trials [3]. To this end, 15 mg daily diethylstilbestrol (DES) showed a 30% response rate including some complete responses when given to patients with metastatic breast cancer exhaustively treated with anti-hormones [6]. Similarly, patients initially responding but then failing AI treatment benefitted from low (6 mg daily) and high dose E_2_ (30 mg daily) treatment with fewer toxic effects observed at low dose [7]. Notably, the antitumor effect is most effective when preceded by long-term E_2_-deprivation as is naturally the case in women 10 years beyond menopause. Additional supportive evidence comes from the Women's Health Initiative (WHI) trial on the health benefits of postmenopausal hormone replacement therapy where treatment of low concentrations of estrogen alone controlled the onset of breast cancer in epithelia with prior E_2_-deprivation, an effect that was most evident in postmenopausal hysterectomized women in their sixties [8].

The antihormone-resistant breast cancer models MCF-7:5C (5C) and MCF-7:2A (2A) were previously generated through clonal selection following long-term E_2_-deprivation of ER-positive MCF-7 cells [9, 10]. Their adaptation to E_2_-deficiency resulted in a wide range of alterations of stress-related pathways which became evident from global gene expression profiles [11]. As an example, both 5C and 2A cells have elevated basal expression levels of JNK (MAPK8), but not p38 (MAPK14) [12]. Moreover, many genes associated with response to stress, including inflammation (e.g. *TNFRSF11B*, *CXCR4*, *TNF*), oxidative stress (e.g. *APOE*, *GPX2*, *SOD2*), endoplasmic reticulum stress (e.g. *EIF2AK3*, *ATF6*, *ERN1*), as well as other stress-related kinases (e.g. *SGK*) have been altered after E_2_-deprivation in both cell lines [12]. The 5C cells undergo E_2_-induced apoptosis within seven days of E_2_-treatment, whereas 2A cells require two weeks. As 2A cells employ stronger antioxidant defense mechanisms compared to 5C cells, they ultimately require oxidative stress in order to die later in the response to E_2_-treatment [13]. At the cellular level, ERα is the target site for E_2_ to induce apoptosis, which can be completely blocked by 4-hydroxytamoxifen (4-OHT) or ERα knockdown [14, 15]. The accumulation of endoplasmic reticulum stress, oxidative stress, and inflammatory stress prior to E_2_-induced apoptosis [11] can be blocked by glucocorticoids and inhibition of c-Src [14, 16]. In particular, the endoplasmic reticulum functions as a key regulatory site for the cell fate decision after E_2_-treatment in both E_2_-deprived cell lines [12]. E_2_ first induces an unfolded protein response [13, 14] which is followed by reduced protein translation *via* protein kinase-like endoplasmic reticulum kinase (PERK) attenuation [14]. Simultaneously, the folding capacity of the endoplasmic reticulum is increased by activating transcription factor (ATF)-6 and inositol-requiring kinase-1 (IRE1) *via* up-regulation of endoplasmic reticulum chaperones and the endoplasmic reticulum-associated protein degradation (ERAD) machinery [12].

It is important to expand the known biology of AI resistance and E_2_-induced apoptosis to the level of gene regulation. MicroRNAs (miRNAs) have emerged as master regulators of gene expression and already have been proven to be of clinical use [17, 18]. MiRNAs are a family of short (22-24 nucleotides), non-coding, single-stranded RNA molecules that among many different biological processes regulate apoptosis and oncogenesis [19, 20]. In breast cancer numerous miRNAs regulate the ERα and *vice versa.* Examples are reviewed in Klinge et al. [21] and include the ERα regulators miR-22, miR-206, miR-221/−222, miR-18a, miR-18b, miR-193b and miR-302c. miR-221 and miR-222 show low expression in ERα-positive cell lines but are overexpressed in 4-OHT-resistant MCF-7 derivative cells as well as in ER-negative breast cancer [22]. Other miRNAs potentially involved in the development of endocrine resistance include miR-15a and miR-16 which suppress the antiapoptotic Bcl2 [23], miR-342 which re-sensitizes MCF-7 cell derivatives to tamoxifen [24], and miR-301, the blockade of which increases the tamoxifen sensitivity of MCF-7 cells [25]. Despite a growing body of literature on miRNAs in tamoxifen resistance little is known on the relevance of miRNAs in AI resistance [26]. Recently, 78 miRNAs were identified to be differentially expressed between MCF-7 and 2A cells including the ER-regulated let-7c, miR-99a, and miR-125b supporting a putative role in endocrine resistance [27].

Here we use AI-resistant breast cancer cell models and report miRNA candidates and their targeted pathways for ‘E_2_-independent growth’ and ‘E_2_-inducible apoptosis’ phenotypes.

## RESULTS

### miRNAs involved in AI resistance and E_2_-inducible apoptosis phenotypes

**Figure 1:**
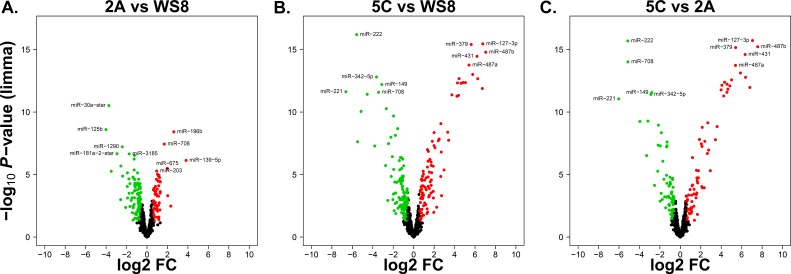
Volcano plots illustrating the differentially expressed miRNAs of breast cancer AI resistance cell models X-axes: log2 FC (fold change); Y-axes: -log_10_ *P*-value from limma analysis. miRNAs with *P*-value < 0.05 and FC > 1.5 are marked in red, with *P*-value < 0.05 and FC < 1/1.5 in green, all others in black. The top five significantly up- down-regulated miRNAs are labeled. Pairwise comparisons: **A.** 2A *versus* WS8. **B.** 5C *versus* WS8. **C**. 5C *versus* 2A.

**Figure 2:**
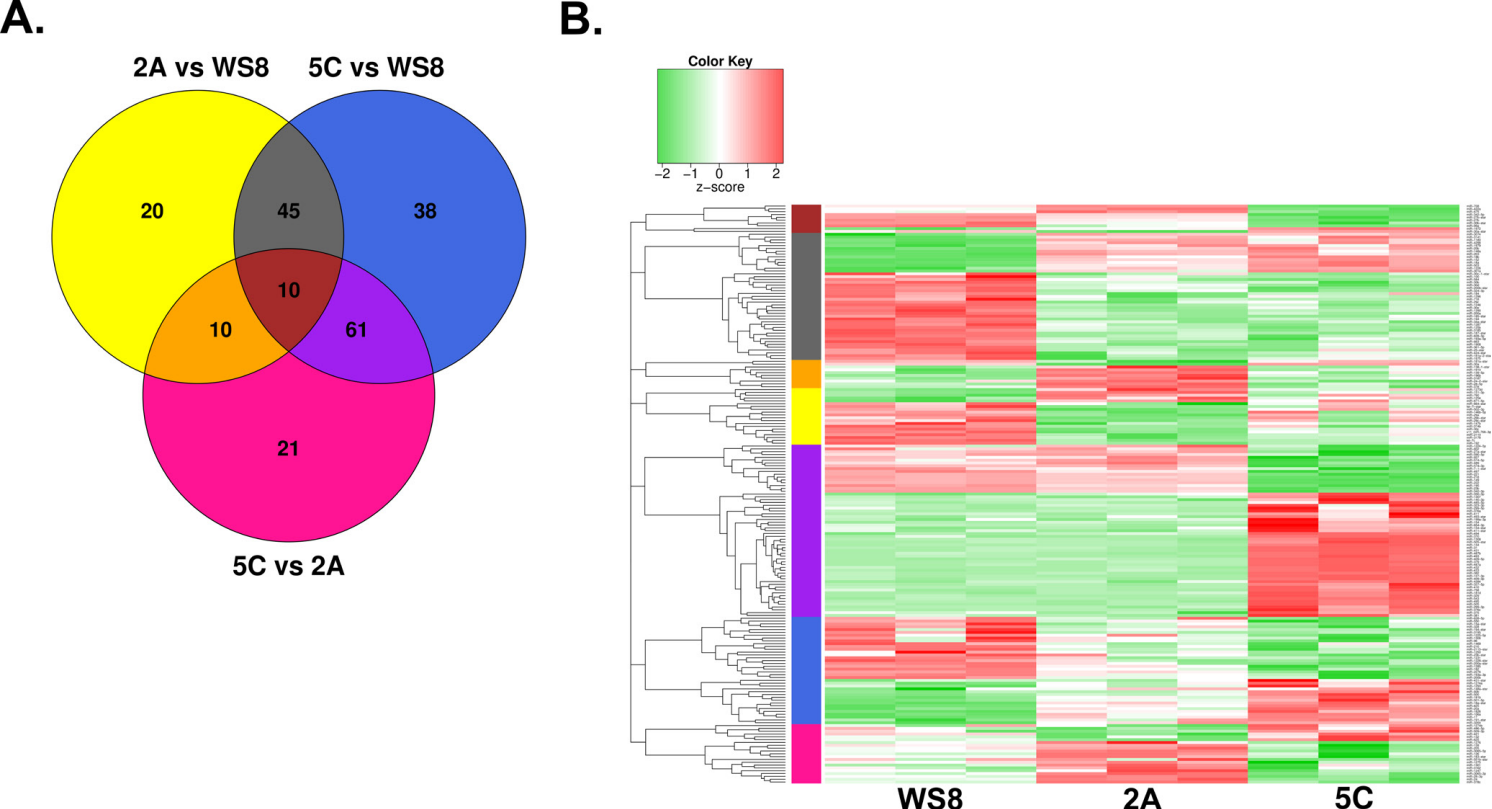
miRNA subsets associated with the phenotypes of AI resistance and E-inducible apoptosis Overall, 205 miRNAs were significantly and relevantly (Holm-adjusted *P*-value < 0.05 and FC > 1.5 or < 1/1.5) differentially expressed in at least one of the three pairwise comparisons (2A *vs* WS8, 5C *vs* WS8, 5C *vs* 2A). **A.** Venn diagram of the three comparisons with numbers of significantly and relevantly differentially expressed miRNAs. There are 55 candidates defining the miRNA set relevant for E_2_-independent growth (grey, red), 99 miRNAs are 5C-specific (blue, purple), and 30 miRNAs are 2A-specific (yellow, orange). The direct comparison of 5C *versus* 2A highlights 21 miRNAs (pink). **B.** Heatmap of the 205 miRNAs comparing WS8, 2A, and 5C with three replicates per cell line. Left color bar matches with colored subsets in A. Within each of the seven color-coded subsets, miRNAs were grouped by complete-linkage hierarchical clustering based on a Pearson correlation distance metric. Corresponding dendrograms are shown.

Top up- and down-regulated miRNA candidates were obtained from the pairwise comparisons 2A *versus* WS8, 5C *versus* WS8, and 5C *versus* 2A (Figure 1). We identified 85 relevantly differentially and significantly expressed miRNAs when comparing 2A *versus* WS8 (FC > 1.5 or < 1/1.5, *P* < 0.05) and 154 miRNAs for 5C *versus* WS8 (Supplementary Tables 1 and 2). Here, 2A cells revealed 31 upregulated miRNAs (top candidates: miR-196b, -708, -139-5p, -675, and -203), and 54 down-regulated miRNAs (top candidates: miR-30a-star, -125b, -1290, -181a-2-star, and -3185) (Figure 1A). 5C cells revealed 76 upregulated miRNAs (top candidates: miR-127-3p, -379, -487b, -431, and -487a), and 78 down-regulated miRNAs (top candidates: miR-222, -342-5p, -149, -221, and -708) (Figure 1B). The 5C *versus* 2A comparison revealed 102 significantly and relevantly differentially expressed miRNAs (Supplementary Table 3) of which 54 are up- and 48 down-regulated matching the top five 5C *versus* WS8 candidates (Figure 1C). The overlap between the 2A *versus* WS8 comparison and the 5C *versus* WS8 comparison disclosed a set of 55 miRNAs that could be relevant for E_2_-independent growth (Figure 2A). Overall, 30 miRNAs are specifically deregulated in 2A *versus* WS8, 99 in 5C *versus* WS8 and 21 additional candidates resulted from the direct comparison (Figure 2A and 2B). In total, 205 miRNAs were significantly and relevantly differentially expressed in at least one of the three pairwise comparisons (2A *vs* WS8, 5C *vs* WS8, 5C *vs* 2A). Figure 2B illustrates the relative expression levels (z-scores) for each of these miRNAs, ordered according to the seven subsets displayed in Figure 2A.

A summary of the miRNAs, which were differentially expressed in at least one of the three pairwise comparisons is given in Table 1A and 1B; for the purpose of reduction, only miRNAs with a mean normalized log2 signal of > 4 and FC > 2 and < ½ are enlisted. The overlapping candidates of both AI resistance models include miR-18a, -18b and -301a (upregulated) and miR-125b, -99a, -27b*, -27b, -342-5p, -708, -181a-2*, -30b, -30b*, -30d, -30e, and -34a* (downregulated), (Table 1A). The 2A-specific miRNAs include miR-139-5p, and -671-5p (upregulated), and miR-29a, -374b, -181a*, and -30a (downregulated), (Table 1B). The 5C-specific miRNAs include miR-18a*, -31, -505, -505*, -375, and -181d (upregulated), and miR-221/222, -218, -149, -342-3p, -23b, -574-3p, -574-5p, -328, -210, -200a*, and -200b (downregulated) (Table 1B). Other important 5C candidates include numerous up-regulated miRNAs from the DLK1-DIO3 locus on Chr. 14q32.31 (Table 1B, underlined).

**Table 1A:** miRNAs sets differentially expressed in AI-resistant cell lines

miRNA	Fold change>^a^	Holm-adj. P^b^	Mean log2 signal^c^ (5C)	Mean log2 signal^c^ (2A)	Mean log2 signal^c^ (WS8)
**Common elements in 5C ***vs*** WS8 and 2A ***vs*** WS8**					
hsa-miR-3074	3.82	1.63E-02	4.64	4.74	2.71
hsa-miR-1226	3.28	1.24E-04	6.04	5.62	4.32
hsa-miR-503	3.26	6.88E-05	7.48	6.92	5.78
hsa-miR-18a	3.03	4.71E-05	10.42	9.95	8.83
hsa-miR-18b	2.90	3.51E-04	7.80	7.25	6.26
hsa-miR-301a	2.71	1.30E-05	9.45	8.94	8.02
hsa-miR-152	2.66	6.01E-05	9.77	9.47	8.36
hsa-miR-1979	2.33	8.28E-04	7.96	7.96	6.74
hsa-miR-148a	2.12	1.13E-03	8.03	8.12	6.94
hsa-miR-125b	1/35.27	1.64E-08	0.93	2.06	6.07
hsa-miR-1246	1/5.77	2.36E-02	5.33	5.28	7.86
hsa-miR-3185	1/4.68	1.33E-06	4.07	4.55	6.30
hsa-miR-181a-2*	1/3.62	1.47E-03	2.99	1.92	4.85
hsa-miR-1296	1/3.36	7.47E-03	2.29	1.65	4.04
hsa-miR-628-3p	1/2.36	1.90E-03	4.86	4.94	6.10
hsa-miR-324-3p	1/2.31	1.81E-03	6.07	6.41	7.28
hsa-miR-361-5p	1/2.28	3.63E-05	10.26	10.22	11.45
hsa-miR-30b	1/2.24	1.96E-04	9.14	9.40	10.30
hsa-miR-193a-5p	1/2.17	6.64E-04	7.07	7.23	8.19
hsa-miR-663	1/2.16	2.96E-03	6.50	6.43	7.61
hsa-miR-1908	1/2.13	8.83E-04	6.89	6.73	7.98
hsa-miR-30e	1/2.13	4.41E-03	6.14	5.88	7.23
hsa-miR-1287	1/2.12	1.21E-03	5.19	5.35	6.27
hsa-miR-34a*	1/2.05	6.42E-04	4.08	4.20	5.11
hsa-miR-30d	1/2.02	6.64E-04	8.73	9.10	9.74
hsa-miR-1975	1/1.69	3.33E-02	10.02	9.57	10.77
**Common elements in 5C ***vs*** WS8, 2A ***vs*** WS8 and 5C ***vs*** 2A**					
hsa-miR-1972	5.64	1.81E-06	7.28	6.07	4.79
hsa-miR-30a*	1.88	1.32E-02	5.42	0.78	4.51
hsa-miR-99a	1/44.62	2.70E-06	0.82	2.80	6.30
hsa-miR-27b*	1/23.35	9.09E-10	1.75	4.92	6.29
hsa-miR-342-5p	1/12.43	8.82E-11	5.41	8.30	9.05
hsa-miR-708	1/10.79	6.63E-10	1.77	6.89	5.20
hsa-miR-30b*	1/5.50	3.74E-06	3.74	5.05	6.20
hsa-miR-27b	1/3.91	3.64E-08	8.62	9.92	10.59
hsa-miR-675	1/3.88	2.30E-04	1.28	5.23	3.24
hsa-miR-422a	1/1.58	5.09E-03	6.87	8.22	7.53
**Elements only in 5C ***vs*** 2A**					
hsa-miR-3065-5p	1/4.63	4.63E-04	4.31	6.52	5.32
hsa-miR-3065-3p	1/3.65	1.39E-02	3.71	5.57	4.32
hsa-miR-378c	1/2.10	4.25E-03	7.87	8.94	8.20

Listed are all miRNAs which fulfill the following criteria for at least one of the three pairwise comparisons (5C *vs* WS8, 2A *vs* WS8, 5C *vs* 2A): (a) fold change > 2 or < ½, (b) Holm-adjusted P < 0.05, and (c) Mean log2 signal > 4 in at least one of the two cell lines being compared, bold: comparison for which respective FC and *P*-values are given.

**Table 1B:** miRNAs sets differentially expressed in AI-resistant cell lines

miRNA	Fold change^a^	Holm-adj. P^b^	Mean log2 signal^c^ (5C)	Mean log2 signal^c^ (2A)	Mean log2 signal^c^ (WS8)
**Elements only in 2A ***vs*** WS8**					
hsa-miR-1273d	2.43	1.79E-02	3.37	4.02	2.74
hsa-miR-671-5p	2.18	2.65E-02	7.80	8.11	6.98
hsa-miR-374b	1/2.77	3.29E-02	5.49	5.17	6.64
hsa-miR-29a	1/2.31	6.76E-03	8.17	7.50	8.70
**Common elements in 2A ***vs*** WS8 and 5C ***vs*** 2A**					
hsa-miR-139-5p	14.17	3.05E-04	3.06	5.59	1.76
hsa-miR-1910	2.11	4.15E-03	4.44	5.31	4.23
hsa-miR-181a-star	1/3.78	1.67E-03	5.34	3.58	5.49
hsa-miR-30a	1/3.73	3.29E-02	7.95	5.61	7.51
**Elements only in 5C ***vs*** WS8**					
hsa-miR-1274a	6.62	1.16E-02	4.77	3.10	2.05
hsa-miR-18a*	2.18	3.27E-03	5.70	5.34	4.57
hsa-miR-328	1/4.59	6.64E-04	2.29	3.23	4.49
hsa-miR-1231	1/4.07	2.05E-02	2.70	3.29	4.72
hsa-miR-200a*	1/2.51	9.84E-04	5.04	5.52	6.37
hsa-miR-1285	1/2.36	1.87E-02	4.23	5.01	5.47
hsa-miR-210	1/2.14	2.95E-03	6.93	7.29	8.03
hsa-miR-550	1/2.13	2.14E-02	3.76	4.34	4.85
hsa-miR-200b	1/2.12	6.49E-04	8.43	8.89	9.51
**Common elements in 5C ***vs*** WS8 and 5C ***vs***2A**					
hsa-miR-487b	132.73	1.86E-12	8.04	0.47	0.99
hsa-miR-127-3p	108.15	6.00E-13	7.74	0.68	0.99
hsa-miR-432	105.24	3.72E-10	7.74	0.93	1.02
hsa-miR-409-3p	76.90	9.79E-11	7.28	0.90	1.02
hsa-miR-431	73.35	3.15E-12	6.81	0.47	0.61
hsa-miR-382	54.71	6.40E-11	6.29	0.40	0.51
hsa-miR-379	49.44	6.00E-13	6.19	0.78	0.57
hsa-miR-487a	42.78	1.32E-11	5.93	0.53	0.51
hsa-miR-433	33.39	1.60E-10	5.63	0.78	0.57
hsa-miR-31	28.67	1.60E-10	5.98	0.97	1.14
hsa-miR-134	24.79	1.60E-10	5.66	1.09	1.02
hsa-miR-409-5p	21.69	9.79E-11	4.82	0.75	0.38
hsa-miR-495	20.78	1.01E-09	5.20	0.64	0.83
hsa-miR-543	19.49	1.60E-10	4.77	0.45	0.49
hsa-miR-329	18.76	1.12E-09	4.69	0.45	0.46
hsa-miR-493	13.35	9.54E-10	4.92	0.93	1.18
hsa-miR-376c	10.65	2.22E-06	4.05	0.61	0.64
hsa-miR-370	10.03	6.48E-07	4.25	0.65	0.92
hsa-miR-505*	6.32	1.41E-07	8.96	6.27	6.30
hsa-miR-494	6.27	6.64E-04	7.53	5.37	4.88
hsa-miR-505	5.32	2.33E-06	7.96	5.57	5.54
hsa-miR-1308	3.99	6.67E-07	10.22	8.08	8.23
hsa-miR-4284	3.75	1.36E-04	7.48	5.71	5.57
hsa-miR-375	3.33	1.08E-03	4.36	1.71	2.62
hsa-miR-330-3p	2.54	1.56E-04	8.31	7.50	6.97
hsa-miR-181d	2.54	9.66E-06	8.09	6.90	6.75
hsa-miR-221	1/99.01	6.36E-10	2.06	8.08	8.69
hsa-miR-222	1/47.64	2.92E-13	3.16	8.30	8.73
hsa-miR-218	1/14.02	5.31E-06	0.56	3.87	4.37
hsa-miR-149	1/8.66	1.90E-10	6.00	8.80	9.11
hsa-miR-342-3p	1/6.39	1.01E-08	9.87	12.01	12.55
hsa-miR-574-3p	1/3.45	1.04E-06	7.12	8.98	8.91
hsa-miR-23b	1/2.95	3.41E-07	10.76	12.05	12.32
hsa-miR-497	1/2.65	5.54E-05	5.83	6.79	7.24
hsa-miR-489	1/2.56	2.44E-04	5.58	7.53	6.93
hsa-miR-574-5p	1/2.44	9.05E-04	4.96	6.67	6.25
hsa-miR-27a*	1/2.32	1.27E-03	3.62	4.96	4.83
hsa-miR-7-1*	1/2.10	2.75E-03	4.52	5.35	5.59
hsa-miR-195	1/2.09	4.59E-05	9.15	9.91	10.21

Listed are all miRNAs which fulfill the following criteria for at least one of the three pairwise comparisons (5C *vs* WS8, 2A *vs* WS8, 5C *vs* 2A): (a) fold change > 2 or < ½, (b) Holm-adjusted *P* < 0.05, and (c) Mean log2 signal > 4 in at least one of the two cell lines being compared, bold: comparison for which respective FC and P-values are given; miRNAs of the DLK1-DIO3 cluster are underlined.

To distinguish miRNAs associated with E_2_-dependent growth that are altered upon short-term E_2_ deprivation from those relevant for the evolution of long-term changes associated to AI resistance and vulnerability to E_2_-induced apoptosis, we compared miRNA expression profiles of WS8 cells under 72h E_2_-treatment *versus* non-treated WS8 reference control. A total of 131 miRNAs were relevantly differentially and significantly expressed (FC > 1.5 or < 1/1.5, *P* < 0.05) (Supplementary Table 4). In addition, the 184 differentially expressed miRNAs between 5C versus WS8 and 2A versus WS8 contain 71 miRNAs that are altered during E_2_-stimulated growth (Supplementary Figure 1).

We confirmed the findings from our global miRNA profilings in that we compared relative miRNA expression levels of a selection of top candidates including miR-31, -221/222, -127-3p, -409-3p and miR-432-5p between 5C, 2A and WS8 using qRT-PCR (Supplementary Figure 2).

### AI resistance-related miRNAs of chromosomal regions 14q32.31 and 21q21.1 are highly expressed in Luminal A breast cancers

**Figure 3:**
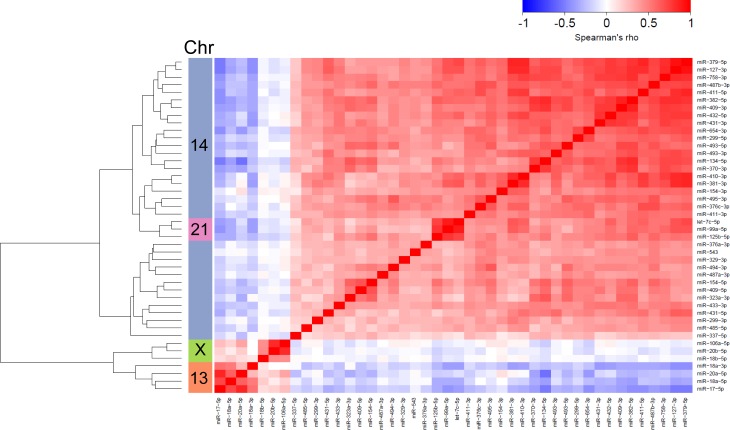
miRNA expression correlations based on TCGA breast cancer data (primary tumors) Hierarchical clustering illustrates intra- and inter-cluster relationships of pairwise comparisons of miRNAs located on chromosomes 14, 21, X and 13. Chromosomal assignments (left color bar) and color-coded Spearman's rho correlation coefficients (blue: low, red: high) are indicated.

We used TCGA miRNA-Seq expression data of 746 breast cancer patients in order to interrogate the putative clinical relevance of miRNAs differentially expressed in the 2A and 5C AI resistance models. Pairwise comparisons of the 205 miRNAs revealed higher correlations between miRNAs located in chromosomal clusters compared to non-clustered miRNAs. Here we focus on those miRNAs that cluster at chromosomal regions 14q32.31, 21q21.1, and 13q31.3. The 14q32.31 mega-cluster comprises among others 34 positively correlated miRNAs overexpressed in 5C (Figure 3). The 21q21.1 cluster includes let-7c, miR-99a and miR-125b, the expression of which is decreased in 2A. In 5C cells, with the exception of let-7c similar changes were observed. Noteworthy, the three miRNAs also showed a strong relationship to miRNAs of the 14q32.31 locus (Figure 3). Furthermore, miRNAs of the 17-92 cluster on chromosome 13q31.3 (miR-17, -18a, -18a* and -20a) and the paralogous 106a-363 cluster on chromosome X (miR-20b, -18b, and -106a) upregulated in 5C show high intra-, but no positive inter-cluster correlations to miRNAs from the 14q32.31 locus, respectively (Figure 3).

**Figure 4:**
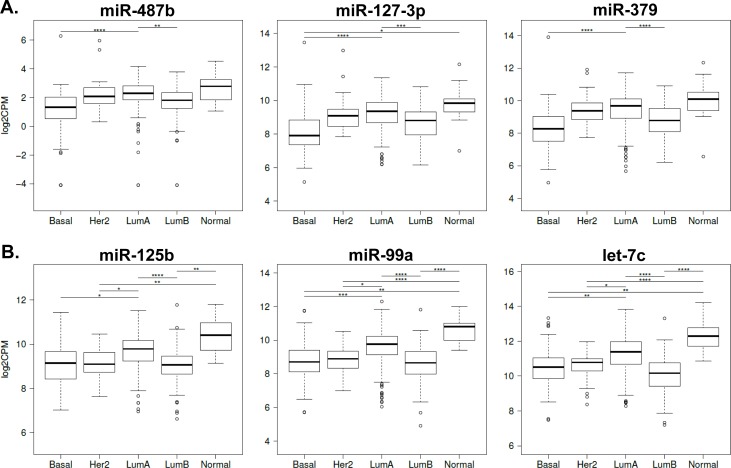

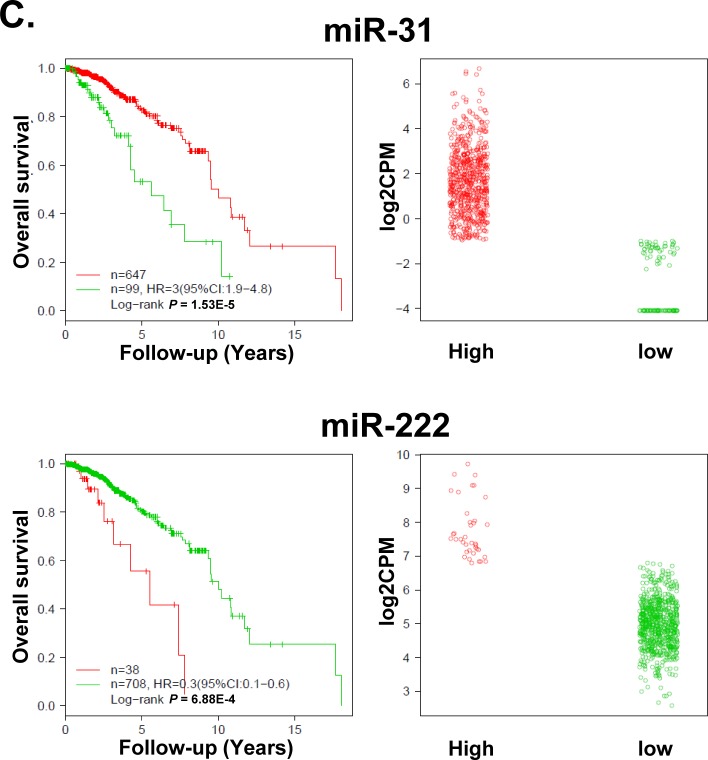
miRNA expression in PAM50-defined breast tumor subtypes from TCGA Box-Whisker plots indicate expression differences between the subgroups Basal-like (Basal, *n* = 86), HER2 positive (Her2, *n* = 43), Luminal A (LumA, *n* = 253), Luminal B (LumB, *n* = 115) and Normal breast-like (Normal, *n* = 16). **A.** Chromosome 14q32.31 miRNAs: miR-487b, miR-127-3p and miR-379. **B.** Chromosome 21q21.1 miRNAs: miR-125b, miR-99a and let-7c. Y-axes: log2CPM (CPM: counts per million). *: *P* < 0.05, **: *P* < 0.01, ***: *P* < 0.001 and ****: *P* < 0.0001. **C.** Kaplan-Meier curves for overall survival stratified by miRNA expression (*n* = 746). Favorable survival is associated with high miR-31 and low miR-222 expression. miRNA expression cut-offs were determined by conditional inference tree models. High (red) and low (green) expression are indicated on the log2CPM scale (CPM: counts per million).

Next, we used the miRNA-Seq data of 513 patients with PAM50 annotation to test for a potential clinical relevance of dysregulated miRNAs (Luminal A, *n* = 253; Luminal B, *n* = 115; Basal-like, *n* = 86; HER2-Enriched, *n* = 43; and Normal breast-like tumors, *n* = 16). Differences between miRNA expression levels in the subtypes were identified for 19 out of the 34 miRNAs at 14q32.31 (Supplementary Figure 3A). For example, the expression of miR-487b, -127-3p and -379 was lowest in Basal-like and Luminal B tumors, however, the Luminal A subgroup shows significantly higher expressions (Figure 4A). Notably, a higher expression of miR-125b, -99a and let-7c (Chr. 21q21.1) was also observed for Luminal A tumors (Figure 4B), a finding that is in accordance with their high correlation to Chr. 14q32.31 miRNAs (Figure 3). AI resistance-related miRNAs at other chromosomal locations also showed differential expression between breast cancer subtypes. These include miR-30a, -375, -342-5p with low expression in Basal-like but high expression in Luminal A tumors. In contrast, we observed high expression of miR-20a, -222, and -18a in Basal-like tumors (Supplementary Figure 3B).

### AI resistance-related miRNAs predict breast cancer outcome

Kaplan-Meier analyses of 746 patients from TCGA identified differences in breast cancer overall survival based on low *versus* high miRNA expression. Altogether, 35 top miRNA candidates (FC > 5 or < 1/5, Table 1A and 1B) and the remaining 16 miRNAs of the 14q32.31 cluster (Supplementary Tables 2 and 3) were analyzed. We identified low expression of miR-31 to be associated with a poor prognosis (HR = 3, 95% CI: 1.9-4.8; *P* = 1.53E-5; Figure 4C), and a low miR-222 expression being associated with a good prognosis (HR = 0.3, 95% CI: 0.1-0.6; *P* = 6.88E-4; Figure 4C). High expression of 7 miRNAs of the 14q32.31 locus (miR-410, -381, -485-5p, -487a, -376c, -411, and -127-3p) indicated a good prognosis, and four others, miR-431, -505*, -493* and -654-3p are associated with the outcome in patients with a Luminal A tumor (*n* = 253) (Supplementary Figure 4A and 4B).

### AI resistance-related genes and key pathways enriched by miRNA sets

**Figure 5:**
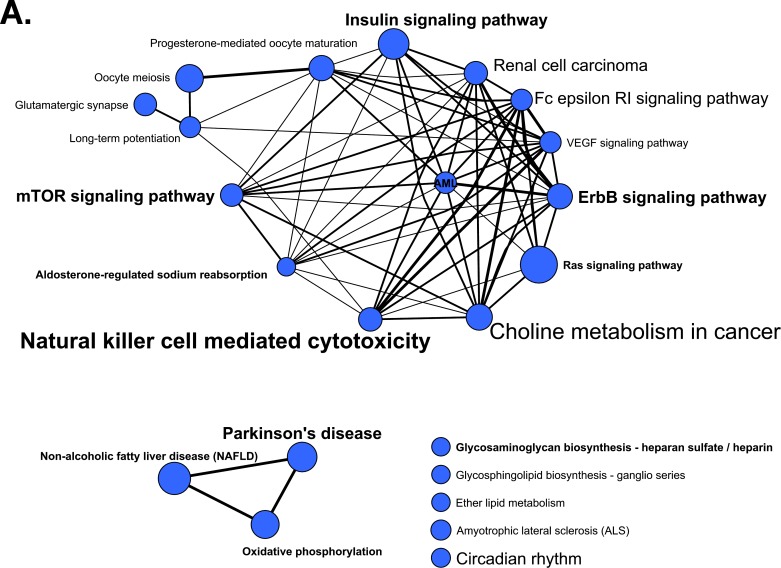

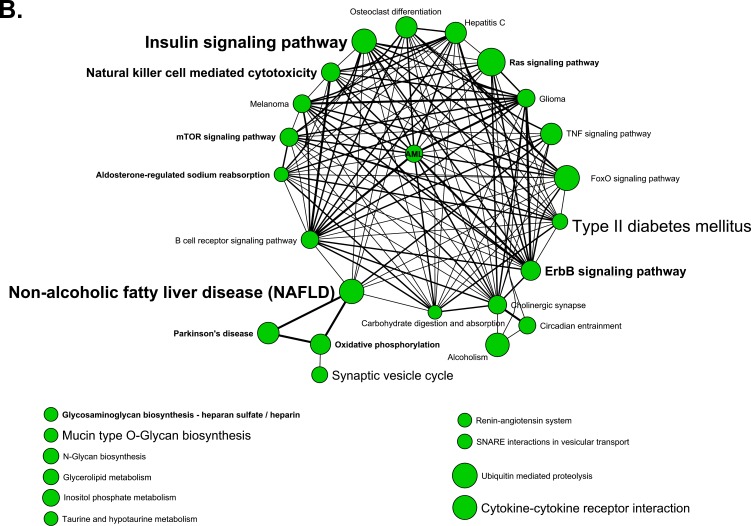

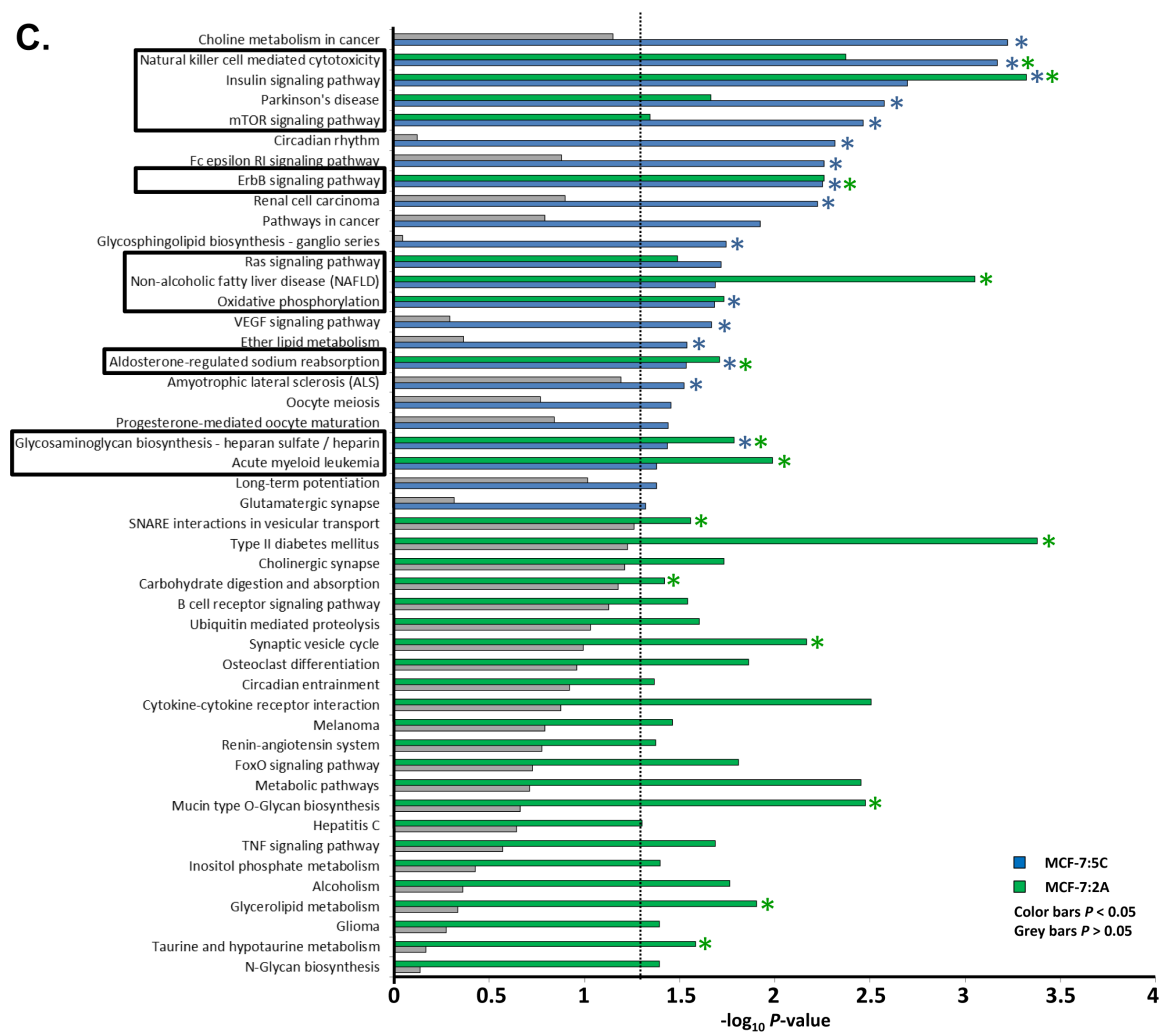

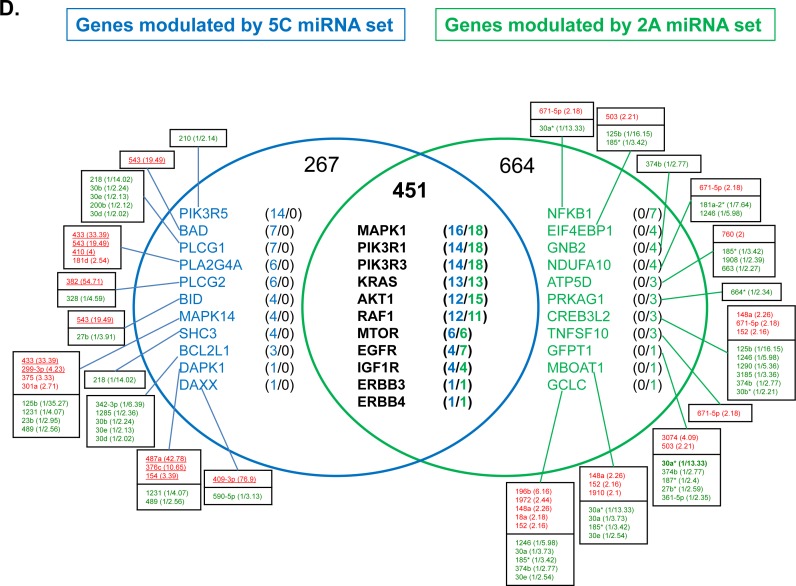
KEGG pathways identified in miRNA functional enrichment analyses Specifically enriched pathways from the entire 5C- and 2A-specific miRNA sets (Tables S1 and S2): **A.** 5C (blue nodes) and **B.** 2A (green nodes). The node size reflects the number of pathway-related genes regulated by the specific miRNA set. The edge weight illustrates the degree of gene overlap between two pathways as measured by the Jaccard index. Pathways highlighted in bold are enriched in both AI resistant cell models. The font size reflects the unadjusted Fisher test P-values from enrichment analysis (*P* < 0.001, large (24 pts); 0.001 ≤ *P* < 0.01, intermediate (18 pts); 0.01 ≤ *P* < 0.05, small (12 pts)). **C.** Enriched pathways of 5C- and 2A-specific miRNA sets. X-axes represent -log_10_ *P*-Value, Y-axes: enriched pathways sorted according to specific pathways enriched in 5C with boxed pathways being enriched in both 5C and 2A models. *P*-value threshold (−log_10_ 0.05) is indicated by the dotted line. Blue and green asterisks mark enriched pathways identified with 5C- and 2A-specific miRNA sets, respectively, based on analyses using permutation tests. *P*-values (Fisher and permutation test) are not corrected for multiple testing. **D.** Venn diagram of the 5C- and 2A- specific pathway-related gene sets extracted from the respective networks (A, B). The intersection represents the total number of genes (451) modulated by both 5C-and 2A-specific miRNA sets. Sections indicate the 5C- and 2A-specific miRNA-modulated genes (5C: 267 genes, 2A: 664 genes). Examples for each subgroup are given in descending order of the number of pathways a gene was detected in both networks. miRNAs with their respective FC (Table 1A and 1B) that interact with and modulate genes in 5C- and 2A cell models are depicted in red (up-regulated) and green (down-regulated). miRNA-mRNA interaction thresholds were defined as CLIP confirmed TargetScan 7.0 in silico predictions (> 50 percentile). miRNAs of the DLK-DIO3 cluster of chromosome 14 are underlined.

To identify pathways relevant in AI resistance, we performed miRNA functional enrichment analyses. First, we ascertained targets of miRNAs that are altered in 5C and 2A generating miRNA-mRNA interaction maps based on CLIP (cross-linking immunoprecipitation)-confirmed TargetScan predictions. Then, miRNAs were linked with KEGG pathways *via* their predicted targets in order to elucidate their functional context. We identified 24 and 34 enriched KEGG pathways for the 5C and 2A-specific miRNA sets, respectively. The networks in Figure 5A and 5B illustrate the overlaps between pathways based on their corresponding gene sets. Eleven pathways are enriched in both 5C and 2A and therefore portraying potential relevance in AI resistance (Figure 5C). These particularly affect, albeit to different degrees, immune response such as the ‘Natural killer cell mediated cytotoxicity’, and growth related pathways such as ‘Insulin signaling’, ‘mTOR signaling’, and ‘ErbB signaling’ (Figure 5A, 5B and 5C). Others affect metabolism such as ‘oxidative phosphorylation’ and ‘glycosaminoglycan biosynthesis-heparan sulfate/heparin’ and disease-related terms including ‘Parkinson's disease’, ‘non-alcoholic fatty liver disease’ and ‘acute myeloid leukemia’ (Figure 5A, 5B and 5C). To account for bias frequently associated with the hypergeometric distribution of miRNA enrichment analyses (standard method) we tested for enrichment using permutation tests (Figure 5C, blue and green asterisks).

Stratification into up- and downregulated miRNA subsets of the 5C and 2A models as well as their pathway overlaps confirms those identified for the entire miRNA sets and enable their assignment (Supplementary Figure 5A, 5B, 5C and 5D). Multiple different metabolic pathways were enriched for the down-regulated miRNA subsets of both 5C and 2A. Proliferation-related pathways appeared to be contributed by both up- and down-regulated miRNA subsets (Supplementary Figure 5A, 5B and 5C).

**Figure 6:**
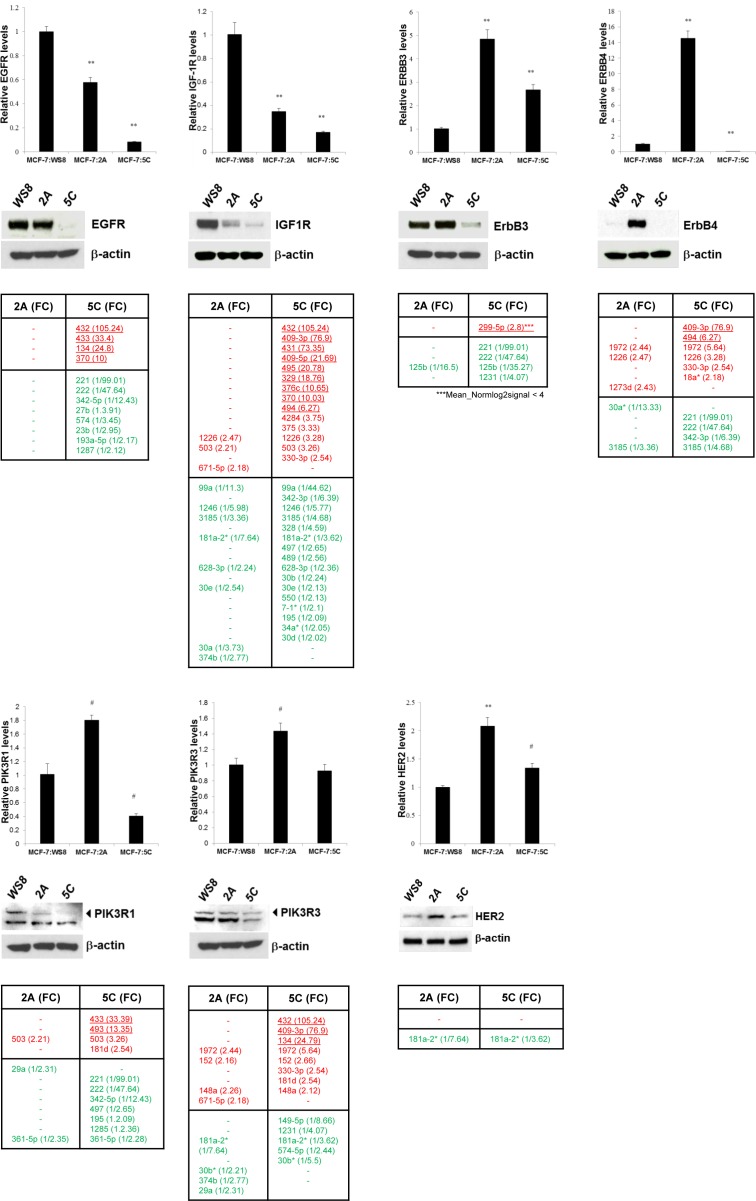
Basal levels of gene and protein expression of key growth regulators in AI resistance models 2A and 5C and their modulatory miRNAs Gene expression levels were quantified by RT-PCR and protein expression levels examined by Western blotting with ß-actin as loading control (WS8: E_2_ growth sensitive control). Up (red) and down (green) regulating miRNAs were obtained from specific miRNA sets given in Table 1A and 1B. miRNA-mRNA interaction thresholds were defined as CLIP confirmed TargetScan 7.0 in silico predictions (> 50 percentile). miRNAs of the DLK-DIO3 cluster of chromosome 14 are underlined. Modulatory effects towards lower protein expression in 5C as compared to 2A are shown for EGFR, ErbB3, ErbB4, IGF1R, PIK3R1, PIK3R3 and HER2. *: *P* < 0.05, #: *P* < 0.01, **: *P* < 0.001 (comparisons to WS8).

To identify AI resistance-relevant genes modulated by both 5C- and 2A-specific miRNA sets we compared their corresponding gene sets obtained from the 5C- and 2A-specific KEGG pathway networks (Figure 5A and 5B). The overlay identifies 451 genes that are modulated by either the 5C- or the 2A-specific miRNA sets. Accordingly, the targeting of 664 genes by the 2A-specific miRNA set and the targeting of 267 genes targeted by the 5C-specific miRNA set may therefore influence the 2A and 5C phenotypes (Figure 5D). Important genes triggered by 5C- and 2A-specific miRNAs include growth factor receptors such as *IGF1R*, *EGFR*, *INSR*, and respective downstream key signal transducers including serine/threonine protein kinases *AKT1, MAPK1, MTOR, RAF1*, as well as other mediators such as *SOS1*, *SOS2*, *JUN*, *PIK3R1*, *KRAS* (Figure 5D). The modulatory impact of miRNAs acting as posttranscriptional regulators cannot be solely captured by RT-PCR of the targeted genes. To identify putative modulatory effects, we therefore investigated the protein levels of key growth factor signal transduction mediators by Western blotting. A strong influence of the 5C-specific miRNA set indicates inhibitory effects on protein expression levels of growth factor receptors. For example, multiple strongly up- and downregulated miRNAs point to a role in the reduction of EGFR, IGF1R, ErbB3 and ErbB4 expression in 5C *versus* 2A cells, respectively (Figure 6). The dominance of the strongly upregulated miRNAs mainly originating from the DLK1-DIO3 cluster (Chr. 14q32.31) underlines their modulatory role on the difference in protein expression which is substantiated by our *in silico* finding of the presence of various dinstinct 3′ UTR CLIP-confirmed interaction sites. Similar findings but with a lesser effect on the protein level were obtained for PIK3R1, PIK3R3 and HER2 (Figure 6) as well as AKT1, MAPK1, KRAS and RAF-1 (Supplementary Figure 6). Minor opposite effects, i.e increased protein expression, have been observed for MTOR (Supplementary Figure 6). miRNAs with postulated major effects on the protein expression of multiple growth related genes are miR-432-5p (e. g. EGFR, IGF1R and PIK3R3) and miR-409-3p (e. g. IGF1R, ERBB4, PIK3R3, AKT1, MAPK1, and KRAS) (Figure 6 and Supplementary Figure 6).

Additional GO term enrichment analyses have been performed with respective gene sets (Figure 5D) to better understand their underlying biological processes. Gene set enrichment analyses (GORILLA) resulted in the identification of major (superordinate) and minor (subordinate) GO terms (Supplementary Figures 7A, 7B, 7C and 8). The gene set targeted by 5C specific miRNAs revealed GO terms referring to signal transduction, protein localization, apoptosis, metabolic processes, and regulation of cell differentiation including Wnt signaling (Supplementary Figure 8). Hence, we observed a dominant role of the regulation of protein phosphorylation and kinase activity including MAPK. Notably, some of the 267 genes targeted by the 5C-specific miRNA set (Figure 5D) enrich for pathways that trigger apoptosis. They include pro-apoptotic genes encoding BAD, BID, DAPK1 and DAXX postulated to be particularly attenuated by miRNAs from the DLK1-DIO3 cluster (e.g. miR-543, miR-487a, miR-409-3p). In contrast, BCL2L1 may be modulated *via* disinhibitory effects of miRNAs such as miR-342-3p and/or members of the miR-30 family (Figure 5D). MAPK14 (p38) is targeted by numerous up- and down-regulated miRNAs and therefore the protein levels may not be affected. The 2A-specific phenotype with 664 specifically targeted genes is characterized by numerous pathways related to metabolism including lipid and amino acids (e.g. glutamine) metabolism. Representative genes include *GFPT1*, *MBOAT1* and *GCLC* (Figure 5D). We postulate that the 2A-specific down-regulated miRNAs such as miR-125b, miR-30a*, miR-374b, miR-181a-2* and miR-1246 may have a dominant effect on the posttranscriptional regulation towards their higher protein expression (Figure 5D). A similar trend pertains to CREB3L2 (Figure 5D), a transcriptional activator involved in the UPR (unfolded protein response) and known to counteract ER stress-induced cell death.

### EGFR protein expression is modulated by miRNAs of the chromosomal region 14q32.31

**Figure 7:**
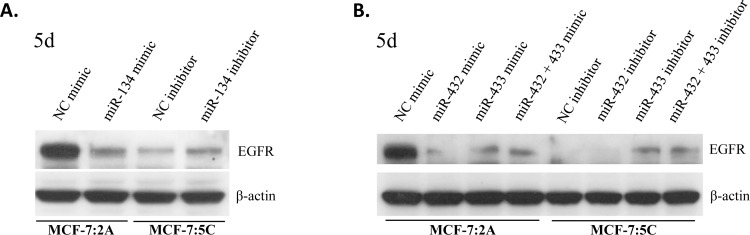
miRNAs of the DLK1-DIO3 cluster downregulate EGFR protein levels in MCF-7:2A (mimics) and upregulate EGFR protein levels in MCF-7:5C (inhibition) **A.** Western blot following mimic and inhibition of miR-134 at day 5. **B.** Western blot following mimic and inhibition of miR-432 and miR-433 as well as simultaneous miR-432 and miR-433 mimic and inhibition at day 5. ß-actin was used as loading control; NC: negative control.

Our *in silico* findings identified distinct CLIP-confirmed interaction sites of the growth factor receptor EGFR 3′UTR sequence to be targeted by miRNAs from both the 5C- and 2A-specific miRNA sets with a dominant role of miRNAs deregulated in 5C (Figure 6). The distinct difference in miRNA panels of 2A and 5C point to a possible role of EGFR and its downstream pathways in the mediation of AI resistance in the 2A model. To determine the impact of upregulated miRNAs from the DLK1-DIO3 cluster on EGFR expression we mimicked the expression of the three top miRNA candidates, miR-432-5p, miR-433-3p and miR-134-5p in 2A cells and inhibited these miRNAs in 5C cells. First data show particularly an influence of miR-134-5p and miR-433-3p on EGFR protein expression. Figure 7A shows a significant downregulation of EGFR protein level by miR-134-5p mimic in 2A cells (not at mRNA level, Supplementary Figure 9A), and a significant upregulation *via* miR-134-5p inhibition in 5C cells after 5 days. Corresponding qRT-PCR controls of miR-134-5p relative levels as well as mRNA expression levels of EGFR are shown in Supplememtary Figure 9A, B. Mimic experiments with miR432-5p, and miR-433-3p as well as simultaneous mimic with both miRNAs in 2A cells showed a clear downregulation of the EGFR protein level (Figure 7B). miRNA inhibiton experiments with single miR-433-3p and simultaneous miR-432-5p and miR-433-3p inhibition in 5C cells show a clear increase of EGFR protein, which appears to be mainly influenced by miR-433-3p (Figure 7B).

## DISCUSSION

We identified more than two hundred up- and down regulated miRNAs relevant to the E_2_-independent growth phenotype inherent to Phase I/II endocrine resistance (Figure 2). We distinguished miRNAs common to both AI resistance models from those specific to 5C and 2A, and investigated their clinical relevance. Thirty-four 5C-specific miRNAs are located at Chr. 14q32.31 (Supplementary Table 2 and 3), a chromosomal region that hosts the largest microRNA cluster in the genome known as the DLK1-DIO3 domain-containing cluster of 54 miRNAs. Together with numerous imprinted genes, antisense and neighbouring long intergenic RNAs they are differentially expressed in several pathologic processes including various cancers [28]. For 34 miRNAs of this cluster, we identified a positive correlation of expression which stresses the existence of common regulatory elements and the dominance of co-transcribed polycistronic primary transcripts (Figure 3). Our finding of high intra-cluster correlations is in accordance with similar findings reported from a large-scale study on miRNA landscapes in breast cancer [29], and highlights their putative implication for AI resistance and vulnerability for E_2_-inducible apoptosis. Notably, we observed high correlations of numerous Chr. 14q32.31 miRNAs with let-7c/miR-99a/miR-125b that are located at another chromosomal cluster 21q21.1 (Figure 3), suggesting their potential cooperation in common regulatory pathways.

The higher expression of numerous miRNAs of the 14q32.31 and 21q21.1 clusters in Luminal A breast tumors compared to Luminal B and Basal like tumors supports the notion of a prominent role of ER as the key histopathological covariate affecting miRNA variability [29]. The high expression of miRNAs miR-210, -17, -18a, and -20a in Basal-like tumors together with our observation of their high expression in AI resistance models suggests their oncogenic potential. The low expression of miRNAs miR-30a, -149, and -342 in the AI resistance models contrasts with a high expression in Luminal A tumors thereby underscoring their role as tumor suppressors during the evolution of AI resistance. Notably, oncogenic and tumor suppressor capacities of these miRNAs have been previously discussed [30–32]. The tumor expression of some miRNAs described herein correlated with breast cancer outcome of patients recorded in TCGA. For example, low expression of miR-31 was associated with a 3-fold increased risk of death. This observation is consistant with the finding of miR-31 as a master regulator of the invasion-metastasis cascade [33]. Its known anti-metastatic function of inhibiting the multistep invasion-metastasis cascade *via* the repression of pro-metastatic targets such as *WASF3* and *ITGB1* [33, 34] aid in the characterization of AI resistance phenotypes. We demonstrated that both genes were only identified in 5C-enriched pathways and respective gene sets (Figure 5A and 5D) and therefore specifically characterize the 5C-specific phenotype. In addition to miR-31, also miR-493-5p from the DLK1-DIO3 locus targets their transcripts and may support attenuation of their protein expression in 5C. Moreover, the capacity of miR-31 to sensitize human breast cells to apoptosis by targeting protein kinase Cε (epsilon) [35] may assist in the understanding of the susceptibility to early E_2_-inducible apoptosis of breast cancer cells particularly in the light of its upregulation in the 5C model. Of note, our CLIP-confirmed TargetScan predictions point to a dominant role of multiple other up-regulated miRNAs in both models with the prevalence of miRNAs from the DLK1-DIO3 locus which may impact on PRKCE levels in 5C.

We showed, that a high expression of miR-222 clearly correlated with non-favorable overall survival of patients recorded in TCGA supporting its role in breast cancer aggressiveness. This finding is in line with recent *in vitro* evidence that miR-221/miR-222 promotes S-phase entry of the cell cycle and cellular migration, and that miR-221 and miR-222 negatively regulate the expression of the tumor suppressor genes, suppressor of cytokine signaling 1 (*SOCS1*) and cyclin-dependent kinase inhibitor 1B (*CDKN1B*) [36]. Stinson et al. [37] reported that miR-221/222 promote a Basal-like phenotype by acting downstream of the oncogenic RAS-RAF-MEK pathway and the triggering of epithelial-to-mesenchymal transition (EMT). Notably, they recognized miR-221/222 as part of the EMT signature as high miR-221/222 levels were correlated with high vimentim and low E-cadherin expression. Our findings of high vimentin (Supplementary Table 5) and low E-cadherin expression in 2A compared to 5C cells supports this role. Moreover, miR-221/222 levels are known to be triggered *via* the activation of the EGFR-(RAS-RAF-MEK) pathway axis [37]. We observed nearly complete loss of EGFR protein in 5C cells and showed that EGFR is the target of miRNAs mainly of the DLK1-DIO3 cluster. We therefore propose that the low EGFR levels in 5C compared to 2A may account for the lowering of miR-221/222 expression thereby suppressing EMT. This may explain the known phenotypic difference with regards to the 5C and 2A EMT status which may have its origin in attenuating effects of miRNAs of the DLK1-DIO3 cluster on EGFR protein expression. Importantly, miR-222 is associated with anti-estrogen resistance [31], and when transfected with miR-221- and/or miR-222, ER-positive cell lines (MCF-7, T47D) develop resistance to tamoxifen, yet their knockdown in ER-negative cells (MDA-MB-468) leads to tamoxifen-induced cell growth arrest and apoptosis. The lack of tamoxifen response in the former can be explained by the negative regulation of ERα protein expression as a consequence of the miR-221/222 targeting of ERα [22]. In this context, the differences in elevation of ERα levels with higher levels detected in 2A compared to 5C (data not shown) may originate from additional modulatory influences of up-regulated miRNAs in 5C. TargetScan predicts multiple high scoring 3′UTR binding sites for miRNAs from the DLK1-DIO3 locus pointing to attenuative functions on ERα protein level in 5C, although one has to consider the complex nature of its regulation *via* other non-posttranscriptional mechanisms. As the mediation of tamoxifen resistance has been further linked to miR-221/222 enclosed in exosomes that act as intercellular bio-messengers [38], we conclude that miR-221/222 is critical for the portrayal of the AI resistance phenotype and susceptibility to E_2_-inducible apoptosis particularly since miR-221/222 is low in 5C cells.

We could not confirm the prognostic evidence for the low expression of let-7c/miR-99a/miR-125b in Luminal A tumors that correlate with poor outcome as recently reported by Bailey *et al.* [27]. They described these miRNAs being present in MCF-7 but not 2A cells and suggest that let-7c and miR-125b inhibit HER2 protein expression in the latter. We observed low expression of these miRNAs in 2A cells, however we were not able to confirm their prognostic character albeit our TCGA sample size was much larger. Interestingly, findings from other AI resistance models demonstrated that miR-125b could be a novel marker of poor prognosis as its overexpression is sufficient to confer resistance to letrozole and anastrozole [39]. Whether or not miR-125b is prognostic in distinct breast cancer subsets remains elusive. In support of a putative clinical relevance of miRNAs of the DLK1-DIO3 cluster, it is noteworthy, that 7 miRNAs (miR-410, -381, -485-5p, -487a, -376c, -411, and -127-3p) were associated with overall survival, with high expression being favorable. Although these clustered miRNAs could be of interest within the context of prognosis, our findings may be preliminary as the median follow-up of 2.2 years in the TCGA breast cancer PAM50 dataset is short. From the patient-based findings, we conclude that the differentially expressed miRNAs identified by us (Table 1A and 1B) bear clinical relevance and should therefore be further investigated as putative treatment targets to subvert AI resistance. In the absence of clinical data linking miRNAs to the development of AI resistance it must be emphasized that miRNAs moving towards cancer therapeutic development are those with sufficient mechanistic data that allow a fairly accurate placement of the miRNA into the disease-related pathways [40].

To elucidate the underlying key cellular processes and pathways, it is important to explore the complex modulatory roles of miRNAs in determining specific AI resistance phenotypes. Aided by miRNA-mRNA networks we enriched for pathways common to the 5C and 2A models and for additional pathways intrinsic to their distinct biology. Both 5C- and 2A-specific miRNA sets enriched for pathways modulating cell growth and proliferation *via* cell surface tyrosine kinase receptors such as insulin-, EGFR-, and ErbB-signaling. Moreover, pathways related to immune response regulation including cytokine signaling *via* JAK/STAT and NFκB were enriched. Our findings support evidence demonstrating that growth factor receptors, such as insulin-like growth factor receptor (IGF-1R), EGFR, and ErbB2, play critical roles in the mediation of endocrine resistance in breast cancer [41–44]. Their functional dominance in the development of resistance is variable and depends on the context of cell lines [42–44]. This is particularly evident from our results in that the clonally selected 5C cells clearly differ from 2A cells in terms of ErbB signaling which we attribute to the distinct modulatory actions of respective miRNA sets on growth pathway-relevant transcipts. Our data show higher protein levels of all ErbB receptors in 2A compared to 5C, for the latter only HER2 is expressed at similar amounts as in the MCF-7:WS8 reference (Figure 6). Therefore we may speculate, that up-regulation of ErbB signaling is used as a compensatory mechanism to continue proliferation in the AI resistant 2A cells, whereas 5C cells seem not to depend on this for survival. These phenotypic differences may account for the stronger resistance to E_2_-inducible apoptosis in the 2A model [11] which is the focus of future studies. On the miRNome level, these differences may be attributed to modulatory effects of multiple miRNAs of the DLK1-DIO3 cluster on key signal transduction molecules in ErbB signaling. First functional data highlight the role of miR-134-5p, miR-432-5p and miR-433-3p in the modulation of EGFR protein levels in 2A and 5C AI resistance models, a finding that is in line with the recently reported direct targeting of the EGFR 3′UTR sequence by miR-134-5p in non-small cell lung cancer cell models [45].

Moreover, it is evident from our results that miRNA modulatory effects on phosphatidylinositol-mediated signaling, Ras signaling, and the MAPK cascade play a dominant role. This is also reflected by the enrichment for protein phosphorylation and kinase activity, in particular MAPK activity, by both 5C- and 2A-specific miRNA sets. Our own observations confirm that IGF-1R is a growth driver in 5C and 2A cells [12, 13]. IGF-1R is regulated by E_2_ in an ER-dependent manner [12] and therefore results in the reduction of total IGF-1R protein in resistance models [44, 46, 47]. However, the cross-talk between IGF-1R and other membrane-associated molecules keeps these resistant cells with higher levels of phosphorylated IGF-1R [44, 46, 47]. The higher levels of phosphorylated IGF-1R are possibly a consequence of an increased modulatory influence of miRNAs on the total amount of protein phosphorylation.

Our enrichment analysis further reveals that the mTOR signaling axis is a main target for miRNAs in both cell lines (Figure 5A and 5B). PI3K/mTOR is a common downstream pathway of growth factor receptors, which is often upregulated in endocrine-resistant breast cancer and proven clinically as a novel treatment target [48, 49]. Notably, the 5C-specific miRNA set additionally enriches for serine/threonine kinase activity and cellular response to stress strengthening our conclusion of an increased modulatory influence on mTOR signaling in 5C. The AKT/mTOR pathway is constitutively activated in AI resistant cell models [39], which is consistent with our pathway enrichment findings. Interestingly, Vilquin *et al*. showed that overexpression of miR-125b activates the AKT/mTOR pathway and increases the capacity to form stem cell-like properties. As miR-125b is one of the top downregulated miRNAs in both of our AI resistance models, we suggest that other miRNAs such as miR-99a [50] and miRNAs of the DLK1-DIO3 locus [51] may interfere with the mTOR pathway during the reprogramming of growth signaling. A suppressive role of the DLK1-DIO3 miRNA mega-cluster on the PI3K-mTOR pathway has been identified in hematopoetic stem cells resulting in a reduction of mitochondrial biosynthesis and metabolic activity and the protection of these cells from excessive production of reactive oxygene species [51]. As miRNAs of this cluster are up-regulated in 5C, future studies will decipher their role in the control of oxidative stress and apoptosis.

A major difference between the AI-resistant 5C and 2A phenotypes is the variable response to oxidative stress triggered by E_2_-treatment. Both cell models respond with endoplasmic reticulum stress and subsequent apoptosis, however at different time points. We attribute the delay of several days of the 2A cells to gluthatione as its basal expression is higher in 2A as compared to 5C cells [13]. Our identified miRNA-mRNA interaction maps revealed potential modulatory effects on the posttranscriptional regulation of enzymes involved in glutathione synthesis such as the γ- glutamylcysteine sythetase (GCLC), as the observed low miR-30a expression in 2A supports this view. Overall, our pathway enrichment analyses suggest a higher metabolic activity for 2A compared to 5C cells. As changes for example in amino acid metabolic pathways might contribute to the complex biology of the early vulnerability of 5C cells to E_2_-induced apoptosis this research topic requires further attention in the future. Importantly, both AI-resistant cell lines exhibit stem cell-like and EMT-like phenotypes, demonstrated by gene expression levels of the putative stem cell markers CD44^high^ and CD24^low^, regression of the cytokeratin expression (*KRT18*), and activation of vimentin expression (Supplementary Table 5). Additionally, Twist, Notch, and Wnt signaling pathways are activated in 5C and 2A cells (Supplementary Table 5). All of these factors have been implicated to be involved in EMT- or stem cell-associated processes which contribute to endocrine resistance. Our pathway enrichment indicated that miRNAs frequently modulate these pathways. Therefore, targeting critical miRNAs to interfere with EMT and to modulate its associated pathways in stem cells has been considered as a potential approach to overcome endocrine resistance [52].

The primary aim of the study was to identify in well characterized cell models of AI resistance the global effects on the modulation of pathways and networks affected by their respective underlying miRNome phenotypes. We defined the intrinsic phenotypes of two AI resistance models, extend available data [27] and provide comprehensive miRNA sets and targeted pathways of 2A and 5C cells. We defined their common and specific features at the miRNA-mRNA interaction level, however changes in miRNA expression are the result of a complex interplay of genomic, transcriptional and post-transcriptional mechanisms which are frequently subtle. Notably, each miRNA can have several targets, some belonging to the same functional network or signaling pathway, and 3′UTR regions of a single gene are frequently targeted by several different miRNAs [39]. Enhancing E_2_-induced apoptosis in AI resistant breast cancer from 30% to 100% responsiveness is an important goal in therapeutics. Understanding the miRNA modulations necessary to trigger apoptosis is an essential first step in the broad application of low dose estrogen as a safe intervention for patients. This extensive database, cross-referenced with clinical databases or functional data, provides the conduit to identify the events that trigger or prevent E_2_-induced apoptosis in AI resistant breast cancer.

## MATERIALS AND METHODS

### Materials

Sources for antibodies for Western blotting are as follows: EGFR antibody was from EMD Millipore (Temecula, CA); IGF-1R antibody was purchased from Santa Cruz (Santa Cruz, CA); ERBB2, ERBB3, ERBB4, MTOR, AKT1, MAPK1, KRAS and RAF-1 antibodies were from cell signaling technology (Beverly, MA); PIK3R1 and PIK3R3 antibodies were obtained from ThermoFisher Scientific (Rockford, IL). *mir*Vana^TM^ miRNA Mimics and Inhibitors for miR-134-3p, 432-5p and 433-3p and respective negative controls (NC) were purchased from ThermoFisher Scientific (Rockford, IL).

### Cell culture

The E_2_-hypersensitive MCF-7:WS8 (WS8) cells were clonally selected from MCF-7 human breast cancer cells and used as the E_2_-dependent reference cell line [53]. The E_2_ deprivation resistant and refractory MCF-7:2A (2A) cells and the sensitive to E_2_-induced apoptosis MCF-7:5C (5C) cells were also clonally selected from MCF-7 cells for maximal growth under long-term estrogen-free conditions. Estrogen-dependent WS8 cells were maintained in fully estrogenized media (phenol red containing RPMI-1640 and 10% whole FBS supplemented with 6 ng/mL insulin, 2 mM glutamine, 100 μM nonessential amino acids, and 100 U penicillin and streptomycin per mL), whereas 5C and 2A cells were maintained in estrogen-free medium (phenol red-free RPMI-1640 plus 10% dextran-coated charcoal-stripped FBS and the same supplements as for fully estrogenized medium) as previously described [11]. Cells were maintained at 37°C in a humidified 5% CO_2_ atmosphere. E_2_-dependent WS8 cells were switched to E_2_-free media (10% SFS) for 3 days, subsequently treated with 0.1% EtOH or 10^−9^ M E_2_ for 3 days and harvested. The DNA content of cells, a measure of proliferation, was determined as previously described [11]. For total RNA extraction for the validation of miRNAs, 5C, 2A and WS8 cells were seeded at 3×10^5^/well in triplicate and harvested after 1 day. WS8 cells were switched to E_2_-free media beforehand for 3 days.

For miRNA mimic and inhibition experiments, 2A and 5C cells were loaded in 6-well plates with a density of 2×10^5^/well in triplicate. After one day, 2A cells were transfected with miR-134-3p, 432-5p, 433-3p mimics and the respective negative control (NC), whereas 5C cells were transfected with respective miRNA inhibitors and negative control (NC) at final concentrations of 30 nM according to the manufacturers specifications. After three days, cells were double transfected with mimics or inhibitors. Cells were harvested 5 days after transfection for qRT-PCR analyses and measurement of protein expression levels of EGFR by Western Blot.

### Western blotting

Proteins were extracted in cell lysis buffer (Cell Signaling Technology, Beverly, MA), supplemented with Proteinase Inhibitor Cocktail Set I and Phosphatase Inhibitor Cocktail Set II (Calbiochem, San Diego, CA). Western blotting was perfomed as previously described [14].

### Quantitative real-time PCR

Quantitative RT-PCR assays were done as previously described [14] using SYBR Green PCR Master Mix from Applied Biosystems (Foster City, CA) on a QuantStudio 6 Flex7900HT Real-time PCR System (Applied Biosystems). For the validation of differentially expressed candidate miRNAs, commercially available TaqMan MicroRNA assays and reagents from ThermoFisher Scientific were used as described in the manufacturers specifications (ThermoFisher Scientific, Rockford, IL, USA). Total RNA was extracted with mirVana™ miRNA Isolation Kit. Briefly, 10 ng total RNA were reverse transcribed and cDNAs were diluted 1:12. For normalization of miRNA qRT-PCR results, RNU44 was used. In parallel, 1μg total RNA was converted to first-strand cDNA using a high capacity cDNA reverse transcription kit (Applied Biosystems) to quantitate expression levels of EGFR.

### Total RNA extraction

Total RNA was isolated using the mirVana^TM^ miRNA Isolation Kit (Applied Biosystems, AM1560) according to the manufacturer's specifications. Briefly, 300 μl Lysis/Binding solution was added to 10^6^ harvested cells and vortexed to obtain a homogenous lysate. RNA samples were checked for purity and integrity using a Nanodrop 1,000 spectrophotometer (Thermo Fisher Scientific, Inc., Waltham, MA, USA) and Agilent 2100 Bioanalyzer (Agilent G2938B) and stored at −80°C until further use.

### MicroRNA microarray

MicroRNA expression profiles were generated utilising GeneChip miRNA2.0 arrays (Affymetrix Inc., Santa Clara, CA, USA) with 1 μg of each total RNA sample according to the manufacturer's instructions. The profiling series included a total of 12 samples. Affymetrix software miRNA QC Tool (Version 1.1.1.0, Affymetrix, 2010) was used for quality control of microarrays and preprocessing of expression data by robust multichip average (RMA). Resulting data were on log2-scale and preprocessed data from all 12 samples (triplicates for WS8, WS8 E_2_ 72h, 2A and 5C) were extracted for this study. The microarray data have been submitted to the GEO online data repository with the accession number GSE79326.

### Statistical analyses and bioinformatics

R-3.2.0 [54] with additional package limma-3.24.10 [55, 56] were used to test the expression data of the three groups (2A, 5C, WS8) against each other. In this investigation, only the 4560 human probe sets were considered. The Holm correction procedure [57] was applied to adjust resulting *P*-values for multiple testing unless otherwise specified. All statistical tests were two-sided unless otherwise specified. Statistical significance was defined as *P* < 0.05. All other statistical analyses including KEGG pathway and GO term enrichment analyses are described in detail in Supplementary materials.

## SUPPLEMENTARY MATERIALS FIGURES


