# Supplementary material for: Profiles of miRNAs matched to biology in aromatase inhibitor resistant breast cancer
Source: Oncotarget. 2016 Sep 17;7(44):71235–54. doi: 10.18632/oncotarget.12103 (PMC5342075; doi:10.18632/oncotarget.12103)
Supplement: Supplementary file 1 [file oncotarget-07-71235-s001.pdf]

Profiles of miRNAs matched to biology in aromatase inhibitor resistant breast cancer

Supplementary Material

MATERIALS AND METHODS

Statistical and Bioinformatic Analyses

Extended statistical analyses were performed with R-3.2.2 including additional packages gplots-2.17.0 [1], partykit-1.0-3 [2], survival-2.38-3 [3] and edgeR-3.10.2 [4]. Open-access miRNA-Seq level 3 isoform quantification data (version 3.1.17.0) and clinical information (version 2.0.28.0) from The Cancer Genome Atlas (TCGA) breast cancer cohort (BRCA) were downloaded from TCGA data portal (https://tcga-data.nci.nih.gov/tcga/) on May 11, 2015. For 746 tumor samples both clinical data and miRNA expression data were available. edgeR was used to calculate the logarithmized counts per million (CPM) values as a measure of expression. To adjust for varying sequencing depth between miRNA-Seq libraries, raw read counts were normalized by edgeR using the trimmed mean of M values approach. By means of sequence identity, miRNAs covered by the Affymetrix miRNA2.0 chip and miRNAs measured by TCGA miRNA-Seq were associated to each other. TCGA breast cancer samples were previously classified using the PAM50 model [5]. PAM50 subtype assignments of 513 tumors were obtained from the Cancer Browser platform (https://genome-cancer.ucsc.edu/) (dataset: TCGA_BRCA_exp_HiSeqV2, version 2015-02-24). KEGG (Kyoto Encyclopedia of Genes and Genomes) pathway data were downloaded from http://www.kegg.jp/kegg/rest/ on June 11, 2015. Full and slim set of Gene Ontology annotations for human were downloaded from http://geneontology.org/page/downloads on September 17, 2015. RefSeq mRNA sequences (genome assembly hg19) were downloaded from UCSC Genome Browser (https://genome.ucsc.edu/cgi-bin/hgTables) on April 29, 2014. Processed CLIP-Seq data provided by the starBase v2.0 web-server (http://starbase.sysu.edu.cn/) were downloaded on April 30, 2014. The miRBase database (version 20) was downloaded on December 14, 2014.

miRNA expression in PAM50 subtypes: Based on raw read counts, miRNAs significantly differentially expressed between PAM50 subtypes were determined using non-parametric Kruskal-Wallis tests as well as pairwise Wilcoxon-Mann-Whitney post-tests.

Survival analyses: Overall survival was used as endpoint in all survival analyses. Associations between survival and miRNA expression was explored using the conditional inference tree framework from the R package partykit. The method recursively partitions the samples in subsets with significantly differing survival functions. The *P*-value cut-off to accept a split was set to 0.1. The significance of the association between groups of samples and overall survival was analyzed by log-rank tests. Holm correction procedure [6] was applied to adjust resulting *P*-values for multiple testing.

Identification of miRNA sets: Putative functions of miRNA sets were identified via in silico functional enrichment analysis of the respective miRNA targets. The population of miRNAs consisted of 1,067 distinct miRNA that were both represented on the array and registered in miRBase version 20. We conducted a transcriptome-wide search on RefSeq mRNA sequences for miRNA targets using the TargetScan software (version 6.2). In order to increase the reliability of target predictions, predicted target sites were overlapped with experimentally identified miRNA-target interaction sites. The starBase v2.0 web-server provides a library of RNA-binding protein interaction sites collected from different CLIP-Seq experiments. Here, we used the subset of 36 CLIP-Seq experiments conducted within 11 different AGO-RNA interaction studies. Inclusion criteria for the definition of target sites were: (A) The target site had to be confirmed by at least one CLIP-Seq study and (B) the percentile value calculated by TargetScan to facilitate comparison of target lists of different miRNAs had to be above 75. A gene was considered to be a target of a certain miRNA if at least one of its associated transcripts contained a minimum of one accepted target site of that miRNA.

Over-represented functional categories are usually identified by comparing the observed overlap between category and miRNA target set with the expected overlap estimated from the background gene set. Notably, the definition of the background gene set is of crucial importance. It is well known, that miRNA targets are enriched for certain functions compared to non-miRNA targets [7,8] and therefore, the

background gene set was compiled in such a way to include only predicted miRNA targets. This is critical in order to avoid a bias in the calling of functional terms. The resulting miRNA-gene interaction map included 1,067 miRNAs and 11,795 genes connected via 263,451 interactions.

KEGG pathway analyses: We based our pathway analyses on the number of interactions between miRNAs and pathway genes. The interaction map was filtered to include genes for which KEGG pathway annotations were available. This resulted in a background interaction map of 1,065 miRNAs, 4,330 genes and 99,246 interactions. Fisher's exact test was used to test if the number of observed interactions between genes of a certain pathway and a miRNA set significantly exceeded the number of expected interactions as estimated from the background interaction map. Additionally, we applied an approach in line with Bleazard *et al*. [9] using permutation tests for the identification of significantly associated pathways. Here, the number of interactions between a miRNA set and a pathway was compared with the number of interactions obtained from a random set of miRNAs of the same size as the input set sampled (without replacement) from the background miRNA set. Sampling was repeated 10,000 times and a permutation *P*-value was calculated using the proportion of random miRNA sets that exhibited equal or more interactions with the pathway genes. The overlap between gene sets from enriched KEGG pathways ($P < 0.05$) were illustrated as graph diagrams executed with yED Graph Editor (http://www.yworks.com/products/yed). Two nodes, representing two different KEGG pathways were connected if their intersection was significantly greater than expected (one-sided Fisher's exact test, correction for multiple testing by Holm's method). Importantly, non-miRNA targets among the pathway genes were not considered for the network construction. The node size refers to the pathway size in terms of the number of miRNA targets. The edge weight illustrates the degree of similarity between two pathways as measured by the Jaccard index. General KEGG pathways of the category "global and overview maps" and "pathways in cancer" were neglected.

Gene Ontology (GO) term enrichment: Over-representation of GO terms were analyzed for the sub-category ‘Biological process’. Target gene lists generated from the overlap between gene sets from significantly enriched KEGG pathways were searched for enriched GO terms using the GORILLA tool

(http://cbl-gorilla.cs.technion.ac.il/). The background gene set was defined as all genes with a GO annotation. GO terms were summarized by removing redundant terms using REVIGO (http://revigo.irb.hr/) using default parameters.

REFERENCES

1. Warnes GR, Bolker B, Bonebakker L, Gentleman R, Liaw WHA, Lumley T, Maechler M, Magnusson A, Moeller S, Schwartz M, Venables B. gplots: Various R programming tools for plotting data: R package version 2.17.0. http://CRAN.R-project.org/package=gplots. 2015.

2. Hothorn T, Zeileis A. partykit: A modular toolkit for recursive partytioning in R. Journal of Machine Learning Research. URL http://EconPapers.RePEc.org/RePEc:inn:wpaper:2014-10. 2015.

3. Therneau TM, Grambsch PM. Modeling survival data: Extending the cox model. New York: Springer; 2000.

4. Robinson MD, McCarthy DJ, Smyth GK. edgeR: a Bioconductor package for differential expression analysis of digital gene expression data. Bioinformatics. 2010; 26: 139-40.

5. The Cancer Genome Atlas Network. Comprehensive molecular portraits of human breast tumours. Nature. 2012; 490: 61-70.

6. Holm S. A simple sequentially rejective multiple test procedure. Scandinavian Journal of Statistics. 1979; 6: 65-70.

7. Cui Q, Yu Z, Pan Y, Purisima EO, Wang E. MicroRNAs preferentially target the genes with high transcriptional regulation complexity. Biochem Biophys Res Commun. 2007; 352: 733-8.

8. Godard P, van EJ. Pathway analysis from lists of microRNAs: common pitfalls and alternative strategy. Nucleic Acids Res. 2015; 43: 3490-7.

9. Bleazard T, Lamb JA, Griffiths-Jones S. Bias in microRNA functional enrichment analysis. Bioinformatics. 2015; 31: 1592-8.

SUPPLEMENTARY TABLES

Supplementary Table 1: miRNAs differentially expressed in MCF-7:2A vs. MCF-7:WS8 cells

Supplementary Table 2: miRNAs differentially expressed in MCF-7:5C vs. MCF-7:WS8 cells

Supplementary Table 3: miRNAs differentially expressed in MCF-7:5C vs. MCF-7:2A cells

Supplementary Table 4: miRNAs differentially expressed in MCF-7:WS8 on E2 treatment (72h). MCF-7:WS8 vs MCF-7:WS8_E2 cells

Supplementary Table 5: Basal expression levels of stem cell-like and EMT-like genes in MCF-7:5C and MCF-7:2A cells

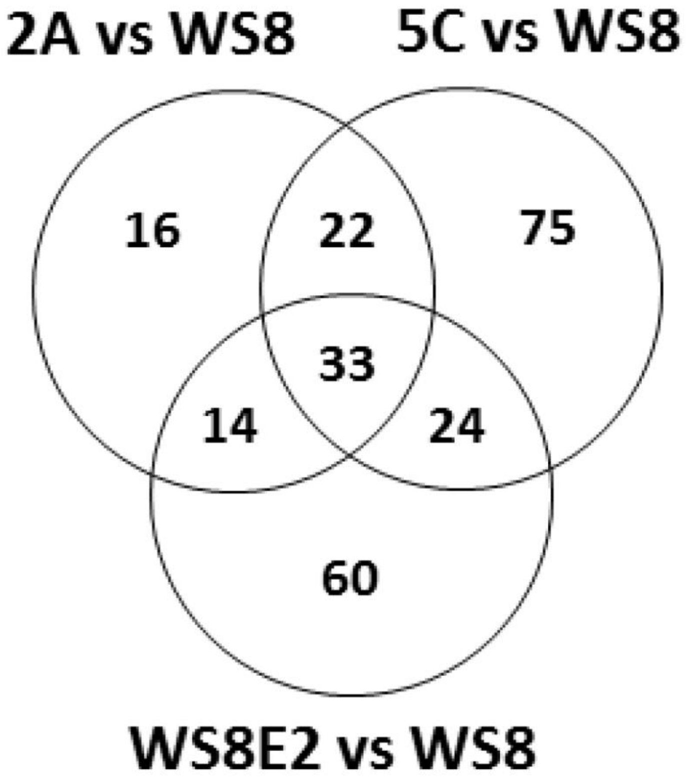

Supplementary Figure 1: miRNAs associated with E2-stimulated growth. Differentially expressed miRNAs of the basal MCF-7:WS8 phenotype following 72h of E_2 exposure (WS8_E_2 versus WS8) are compared to pairwise comparisons of 2A versus WS8 cells and 5C versus WS8 cells to identify growth-related miRNAs. The Venn diagram intersections highlight the 71 WS8 specific miRNAs (14+33+24) subject to changes during E_2 exposure. Thirty-three miRNAs overlap with the basal AI resistance phenotypes 2A and 5C.

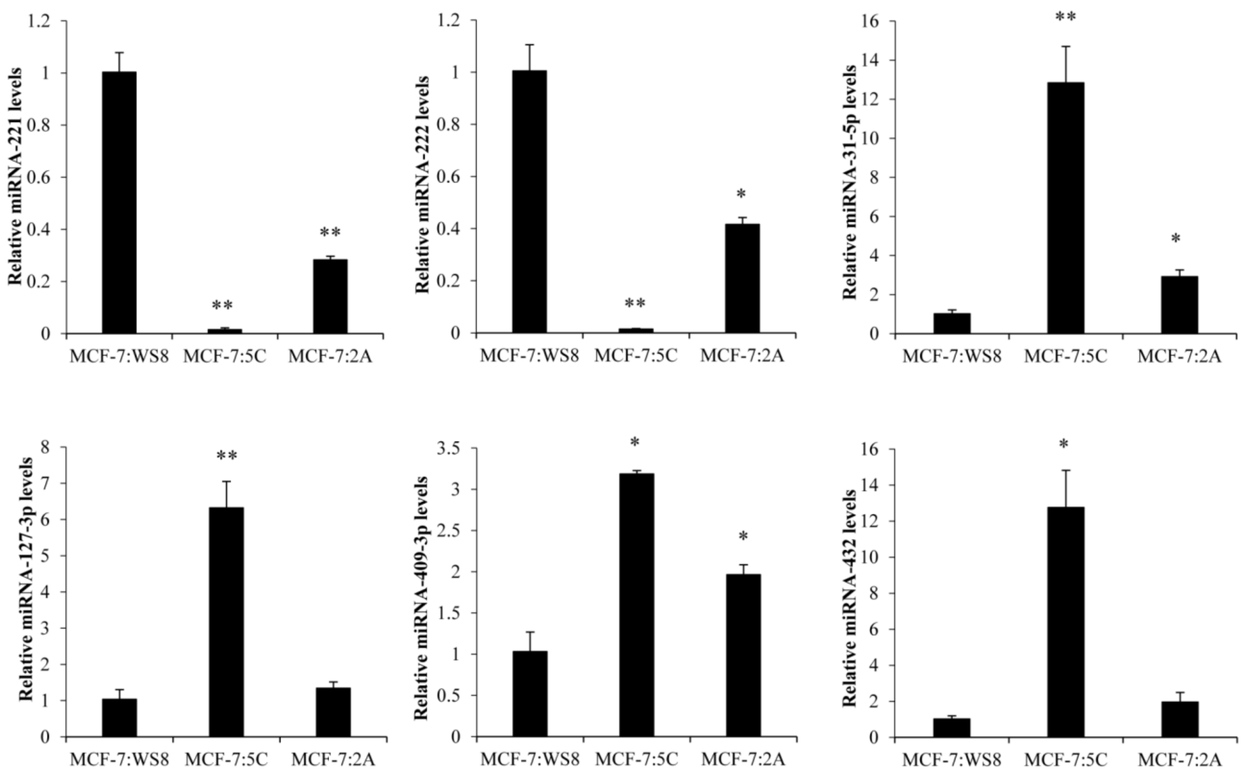

Supplementary Figure 2: Basal levels of expression of candidate miRNAs between the AI-resistant (5C, 2A) and WS8 cells. Relative miRNA expression was quantified by RT-PCR using RNU44 for normalization. Results are shown for miR-221/222, -31, -127-3p, -409-3p and -432-5p with experiments done in triplicates *: $P < 0.05$, **: $P < 0.001$.

A.

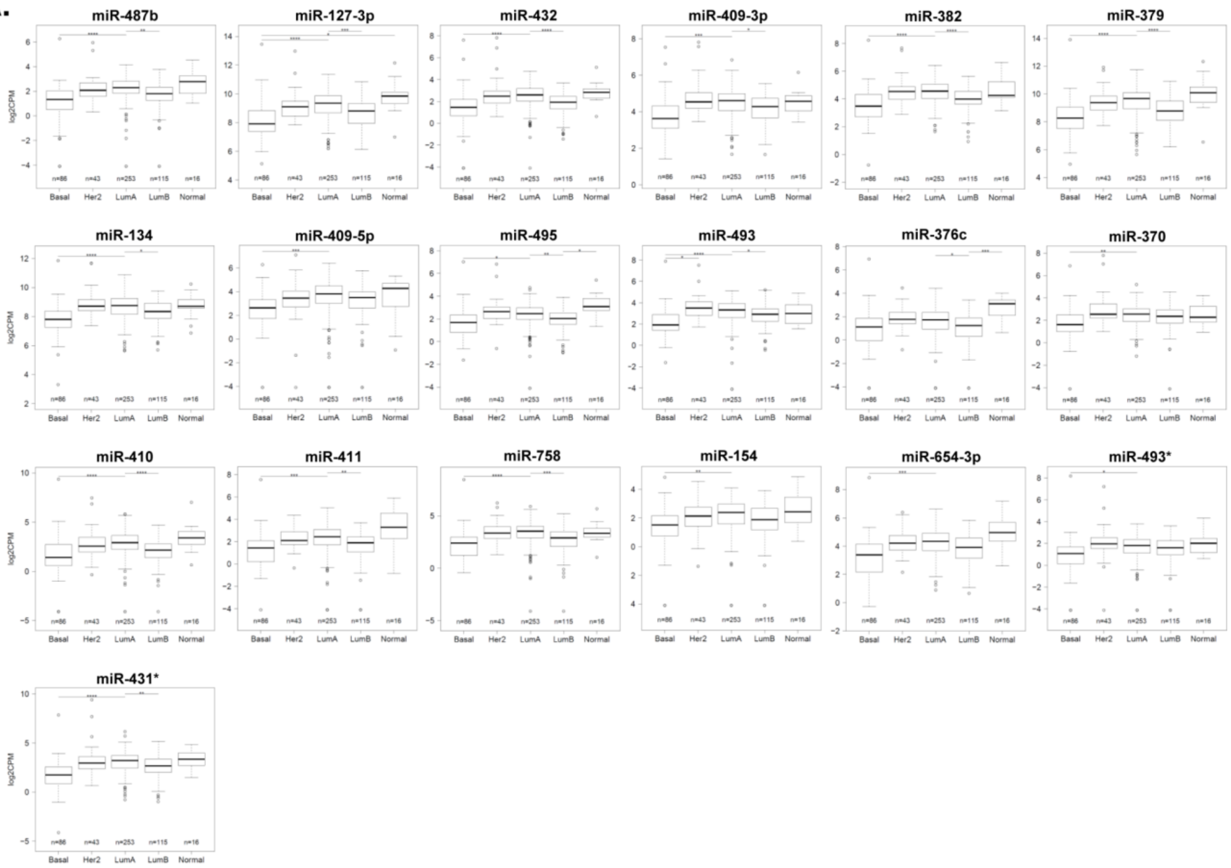
miR-487b
miR-127-3p
miR-432
miR-409-3p
miR-382
miR-379
miR-134
miR-409-5p
miR-495
miR-493
miR-376c
miR-370
miR-410
miR-411
miR-758
miR-154
miR-654-3p
miR-493*
miR-431*
log2CPM
Basal
Her2
LumA
LumB
Normal
n=86
n=43
n=253
n=115
n=16

B.

yellow, orange: 2A specific miRs

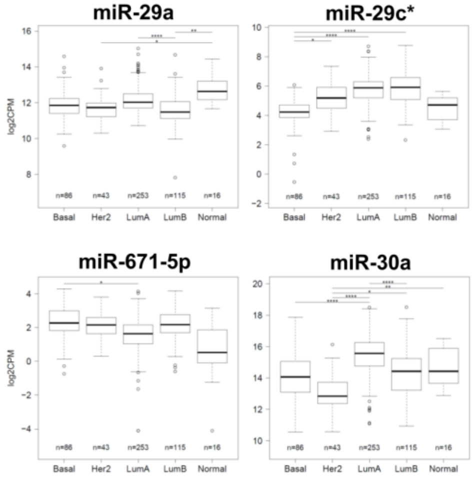

blue, purple: 5C specific miRs

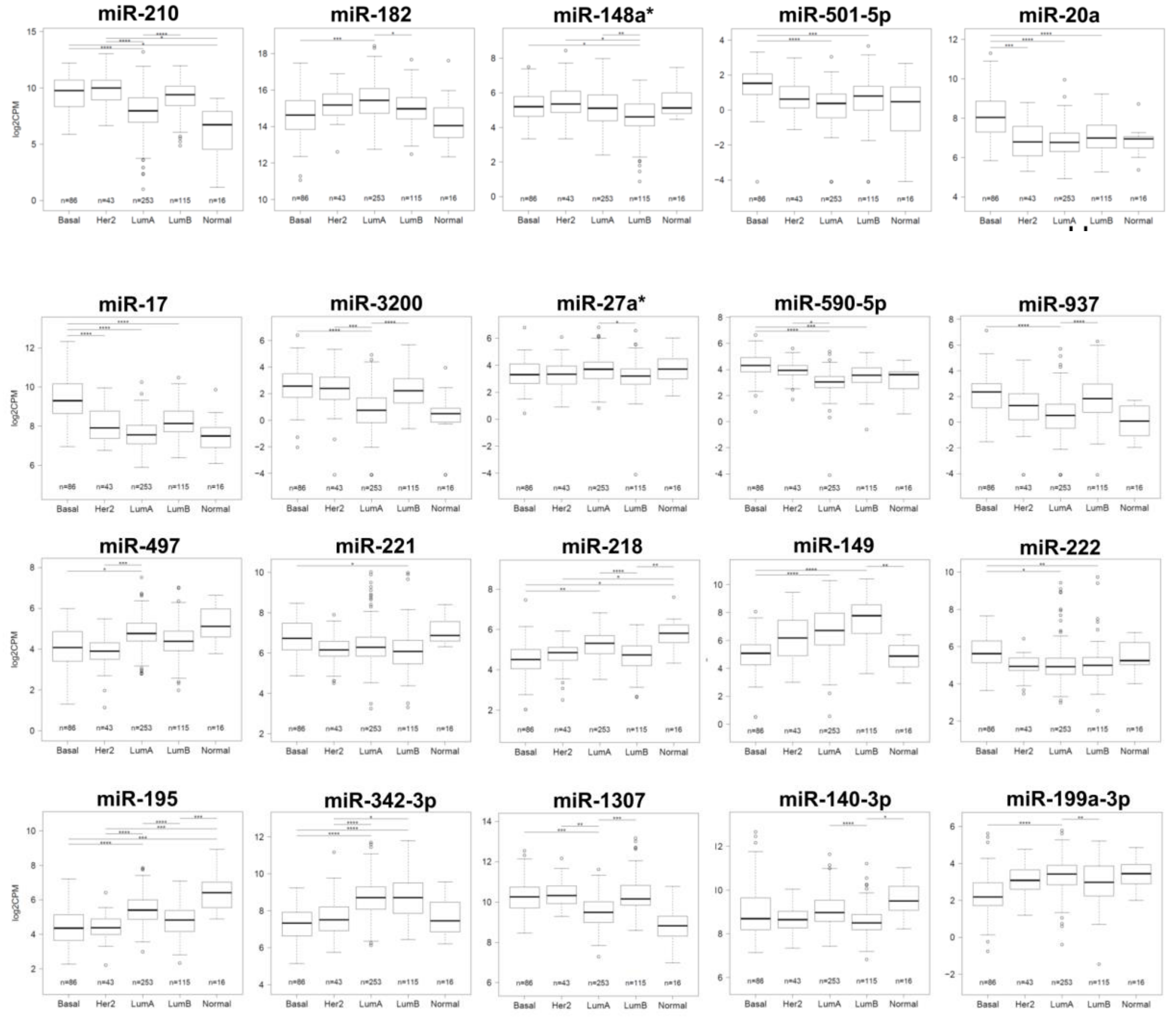

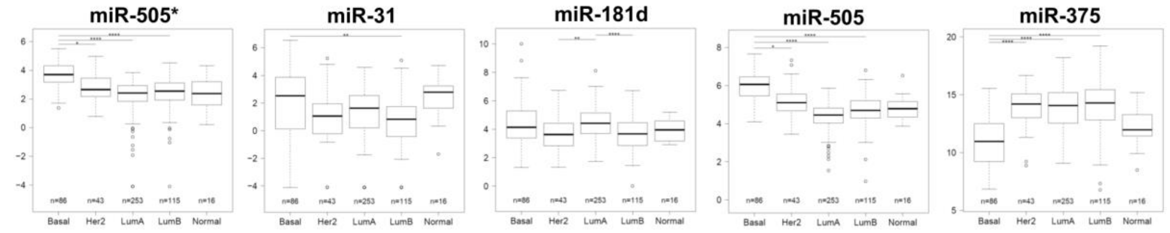

grey, red: 5C and 2A specific miRs:

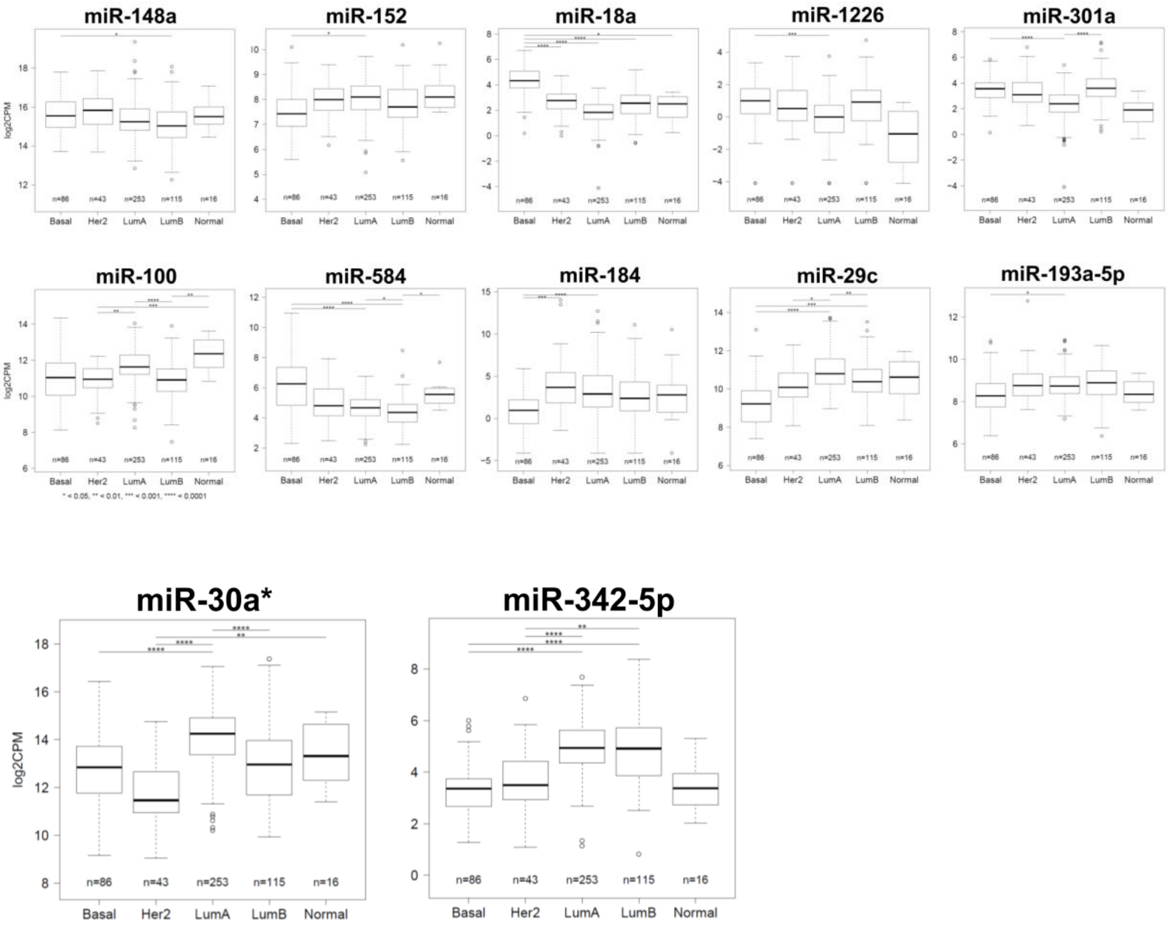

Supplementary Figure 3: miRNA expression in PAM50-defined breast tumor subtypes from TCGA. Box-Whisker plots indicate expression differences between the subgroups Basal-like (Basal, n=86), HER2 positive (Her2, n=43), Luminal A (LumA, n=253), Luminal B (LumB, n=115) and Normal breast-like (Normal, n=16). **A.** Expression levels of chromosome 14q32.31 miRNAs show differences for 19 miRNAs in the various subgroups (Holm-adjusted $P < 0.05$). **B.** Expression levels of the remainder miRNAs in the breast cancer subgroups. Plots are sorted according to the 2A-specific, 5C-specific and the

2A-5C-intersection miRNAs (see color code in Figure 2A and B). Y-axes: log2CPM (CPM: counts per million). *: $P < 0.05$, **: $P < 0.01$, ***: $P < 0.001$ and ****: $P < 0.0001$.

A.

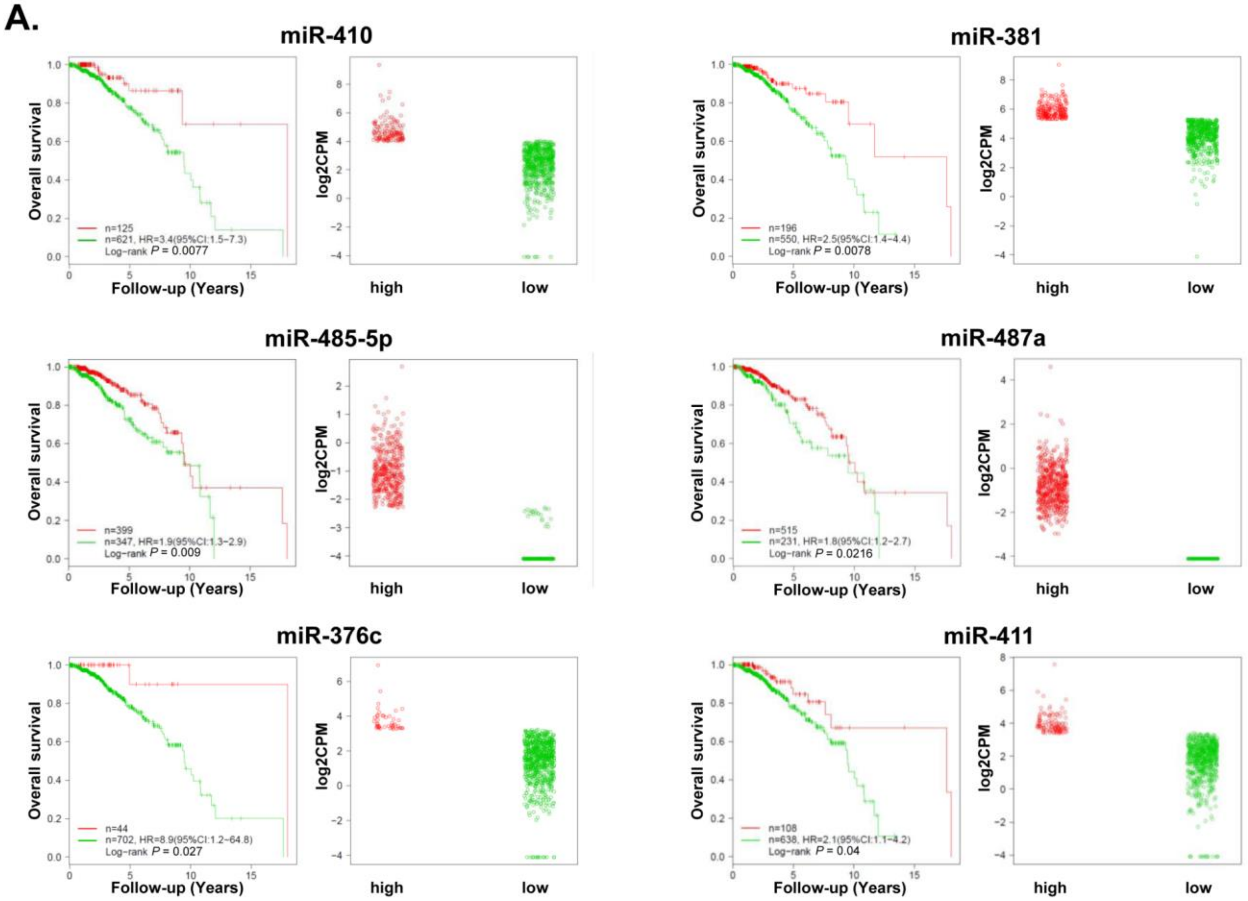

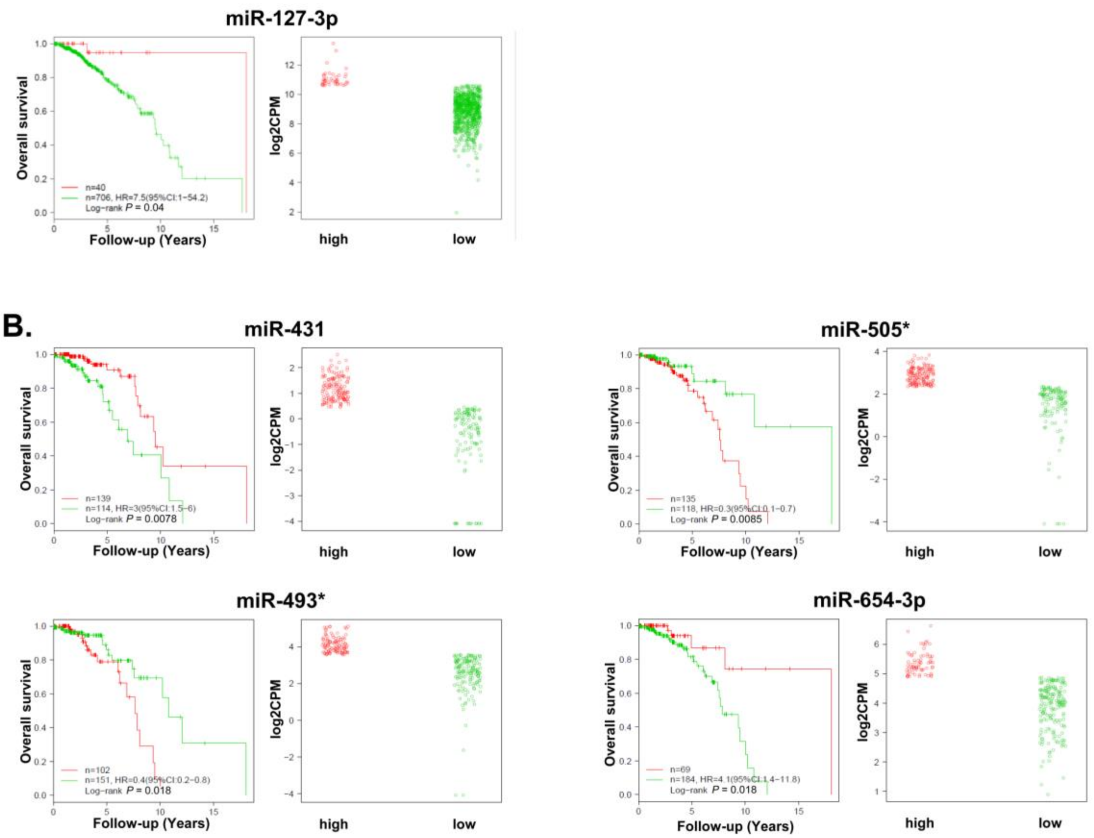

Supplementary Figure 4: Kaplan-Meier curves of overall survival of breast cancer patients of TCGA stratified by miRNA expression. MiRNA expression cut-offs were determined by conditional inference tree models. High (red) and low (green) expression are indicated on the log2CPM scale (CPM: counts per million). **A.** Analyses across all 746 patients showed associations between high expression of miR-410, -381, -485-5p, -487a, -376c, -411, and -127-3p and favorable outcome as compared to low expression, respectively. **B.** Analyses across 253 patients with Luminal A tumors show associations of high expression of miR-431 and -654-3p with favorable outcome as compared to low expression, respectively. In contrast, low expression of miR-493* and -505* was associated with favorable outcome compared to high expression, respectively. Holm correction was applied to adjust *P*-values for multiple testing.

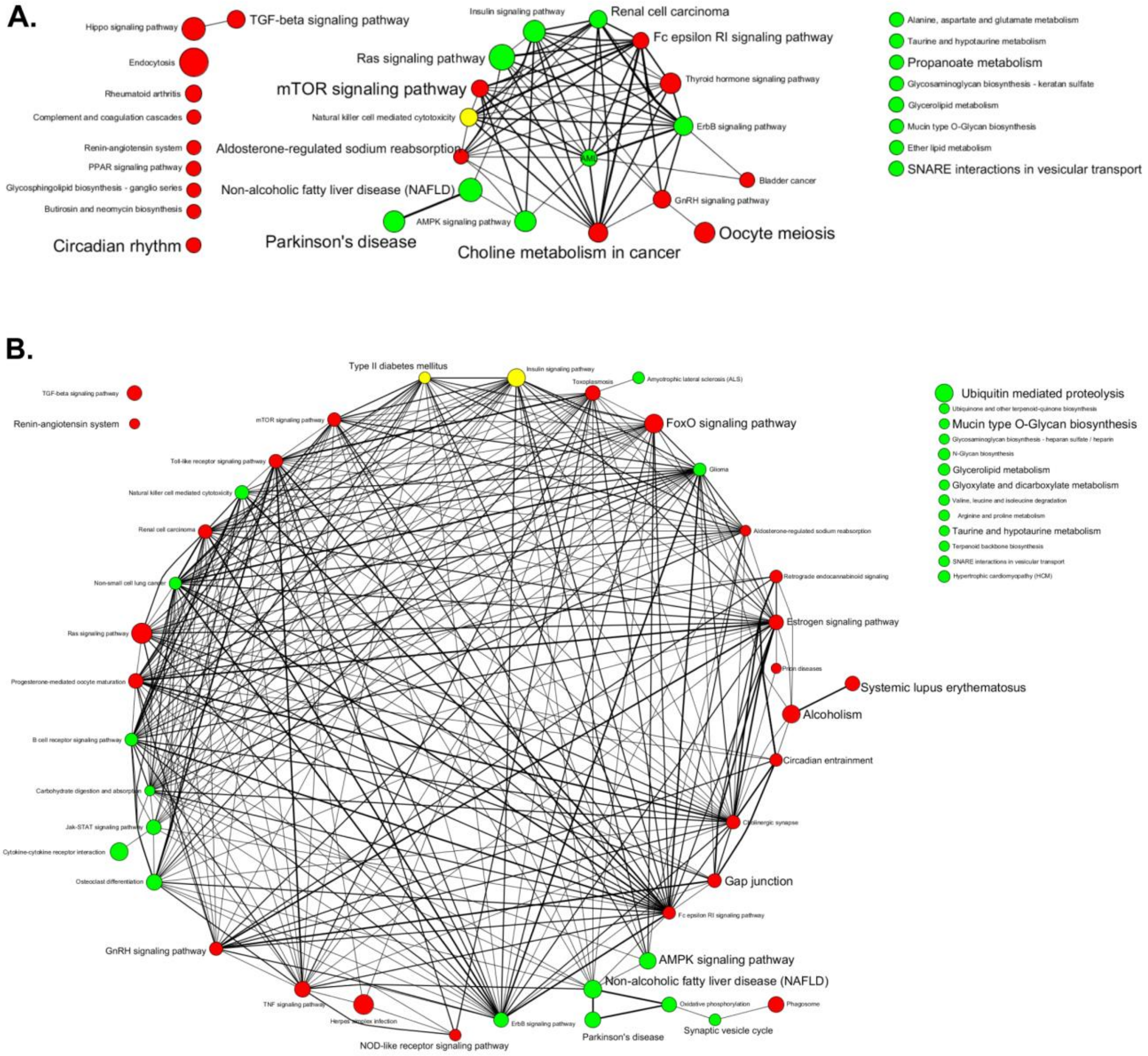

A.
Hippo signaling pathway
TGF-beta signaling pathway
Endocytosis
Rheumatoid arthritis
Complement and coagulation cascades
Renin-angiotensin system
PPAR signaling pathway
Glycosphingolipid biosynthesis - ganglio series
Butirosin and neomycin biosynthesis
Circadian rhythm
Insulin signaling pathway
Renal cell carcinoma
Fc epsilon RI signaling pathway
Ras signaling pathway
mTOR signaling pathway
Thyroid hormone signaling pathway
Natural killer cell mediated cytotoxicity
ErbB signaling pathway
Aldosterone-regulated sodium reabsorption
Bladder cancer
Non-alcoholic fatty liver disease (NAFLD)
GnRH signaling pathway
AMPK signaling pathway
Parkinson's disease
Choline metabolism in cancer
Oocyte meiosis
Alanine, aspartate and glutamate metabolism
Taurine and hypotaurine metabolism
Propanoate metabolism
Glycosaminoglycan biosynthesis - keratan sulfate
Glycerolipid metabolism
Mucin type O-Glycan biosynthesis
Ether lipid metabolism
SNARE interactions in vesicular transport
B.
TGF-beta signaling pathway
Renin-angiotensin system
Type II diabetes mellitus
Insulin signaling pathway
Toxoplasmosis
Amyotrophic lateral sclerosis (ALS)
mTOR signaling pathway
FoxO signaling pathway
Toll-like receptor signaling pathway
Glioma
Natural killer cell mediated cytotoxicity
Renal cell carcinoma
Aldosterone-regulated sodium reabsorption
Non-small cell lung cancer
Retrograde endocannabinoid signaling
Ras signaling pathway
Estrogen signaling pathway
Progesterone-mediated oocyte maturation
Prion diseases
Systemic lupus erythematosus
Alcoholism
B cell receptor signaling pathway
Circadian entrainment
Carbohydrate digestion and absorption
Jak-STAT signaling pathway
Cholinergic synapse
Cytokine-cytokine receptor interaction
Osteoclast differentiation
Gap junction
Fc epsilon RI signaling pathway
GnRH signaling pathway
AMPK signaling pathway
Non-alcoholic fatty liver disease (NAFLD)
TNF signaling pathway
Herpes simplex infection
Oxidative phosphorylation
Phagosome
ErbB signaling pathway
Parkinson's disease
Synaptic vesicle cycle
NOD-like receptor signaling pathway
Ubiquitin mediated proteolysis
Ubiquinone and other terpenoid-quinone biosynthesis
Mucin type O-Glycan biosynthesis
Glycosaminoglycan biosynthesis - heparan sulfate / heparin
N-Glycan biosynthesis
Glycerolipid metabolism
Glyoxylate and dicarboxylate metabolism
Valine, leucine and isoleucine degradation
Arginine and proline metabolism
Taurine and hypotaurine metabolism
Terpenoid backbone biosynthesis
SNARE interactions in vesicular transport
Hypertrophic cardiomyopathy (HCM)

C.

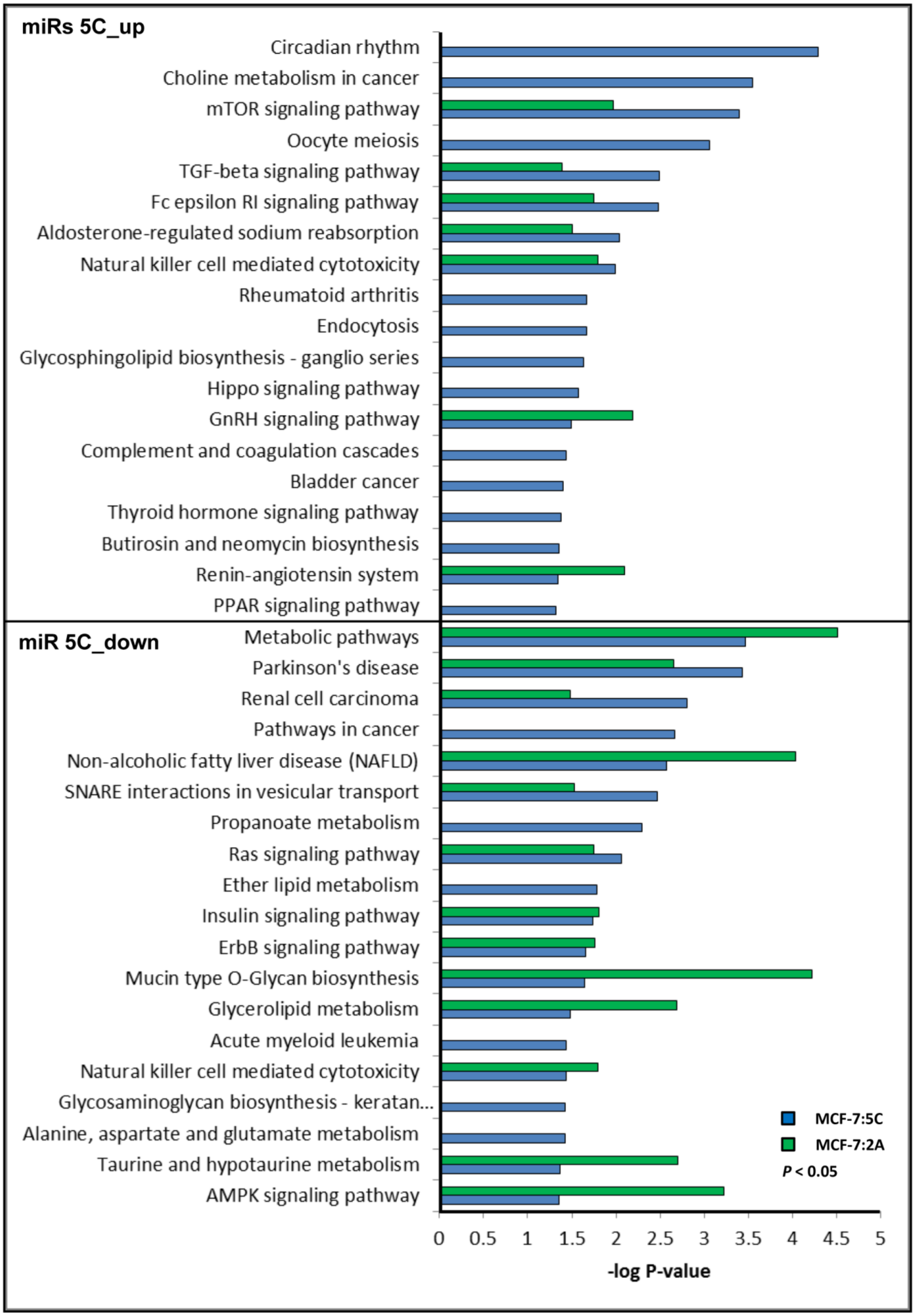
miRs 5C_up
Circadian rhythm
Choline metabolism in cancer
mTOR signaling pathway
Oocyte meiosis
TGF-beta signaling pathway
Fc epsilon RI signaling pathway
Aldosterone-regulated sodium reabsorption
Natural killer cell mediated cytotoxicity
Rheumatoid arthritis
Endocytosis
Glycosphingolipid biosynthesis - ganglio series
Hippo signaling pathway
GnRH signaling pathway
Complement and coagulation cascades
Bladder cancer
Thyroid hormone signaling pathway
Butirosin and neomycin biosynthesis
Renin-angiotensin system
PPAR signaling pathway
miR 5C_down
Metabolic pathways
Parkinson's disease
Renal cell carcinoma
Pathways in cancer
Non-alcoholic fatty liver disease (NAFLD)
SNARE interactions in vesicular transport
Propanoate metabolism
Ras signaling pathway
Ether lipid metabolism
Insulin signaling pathway
ErbB signaling pathway
Mucin type O-Glycan biosynthesis
Glycerolipid metabolism
Acute myeloid leukemia
Natural killer cell mediated cytotoxicity
Glycosaminoglycan biosynthesis - keratan...
Alanine, aspartate and glutamate metabolism
Taurine and hypotaurine metabolism
AMPK signaling pathway
MCF-7:5C
MCF-7:2A
P < 0.05
0
0.5
1
1.5
2
2.5
3
3.5
4
4.5
5
-log P-value

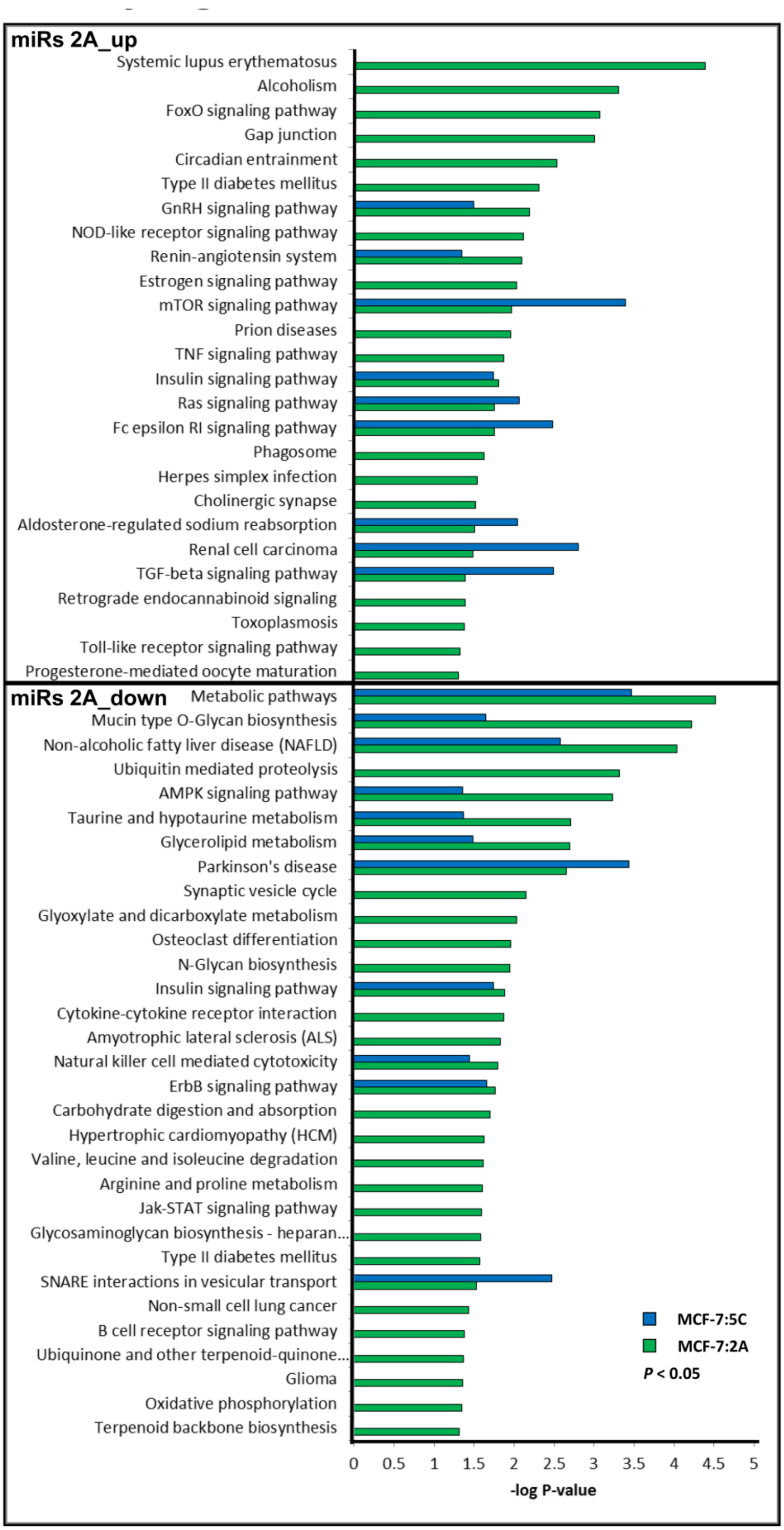

miRs 2A_up
Systemic lupus erythematosus
Alcoholism
FoxO signaling pathway
Gap junction
Circadian entrainment
Type II diabetes mellitus
GnRH signaling pathway
NOD-like receptor signaling pathway
Renin-angiotensin system
Estrogen signaling pathway
mTOR signaling pathway
Prion diseases
TNF signaling pathway
Insulin signaling pathway
Ras signaling pathway
Fc epsilon RI signaling pathway
Phagosome
Herpes simplex infection
Cholinergic synapse
Aldosterone-regulated sodium reabsorption
Renal cell carcinoma
TGF-beta signaling pathway
Retrograde endocannabinoid signaling
Toxoplasmosis
Toll-like receptor signaling pathway
Progesterone-mediated oocyte maturation
miRs 2A_down
Metabolic pathways
Mucin type O-Glycan biosynthesis
Non-alcoholic fatty liver disease (NAFLD)
Ubiquitin mediated proteolysis
AMPK signaling pathway
Taurine and hypotaurine metabolism
Glycerolipid metabolism
Parkinson's disease
Synaptic vesicle cycle
Glyoxylate and dicarboxylate metabolism
Osteoclast differentiation
N-Glycan biosynthesis
Insulin signaling pathway
Cytokine-cytokine receptor interaction
Amyotrophic lateral sclerosis (ALS)
Natural killer cell mediated cytotoxicity
ErbB signaling pathway
Carbohydrate digestion and absorption
Hypertrophic cardiomyopathy (HCM)
Valine, leucine and isoleucine degradation
Arginine and proline metabolism
Jak-STAT signaling pathway
Glycosaminoglycan biosynthesis - heparan…
Type II diabetes mellitus
SNARE interactions in vesicular transport
Non-small cell lung cancer
B cell receptor signaling pathway
Ubiquinone and other terpenoid-quinone…
Glioma
Oxidative phosphorylation
Terpenoid backbone biosynthesis
MCF-7:5C
MCF-7:2A
P < 0.05
0 0.5 1 1.5 2 2.5 3 3.5 4 4.5 5
-log P-value

D.

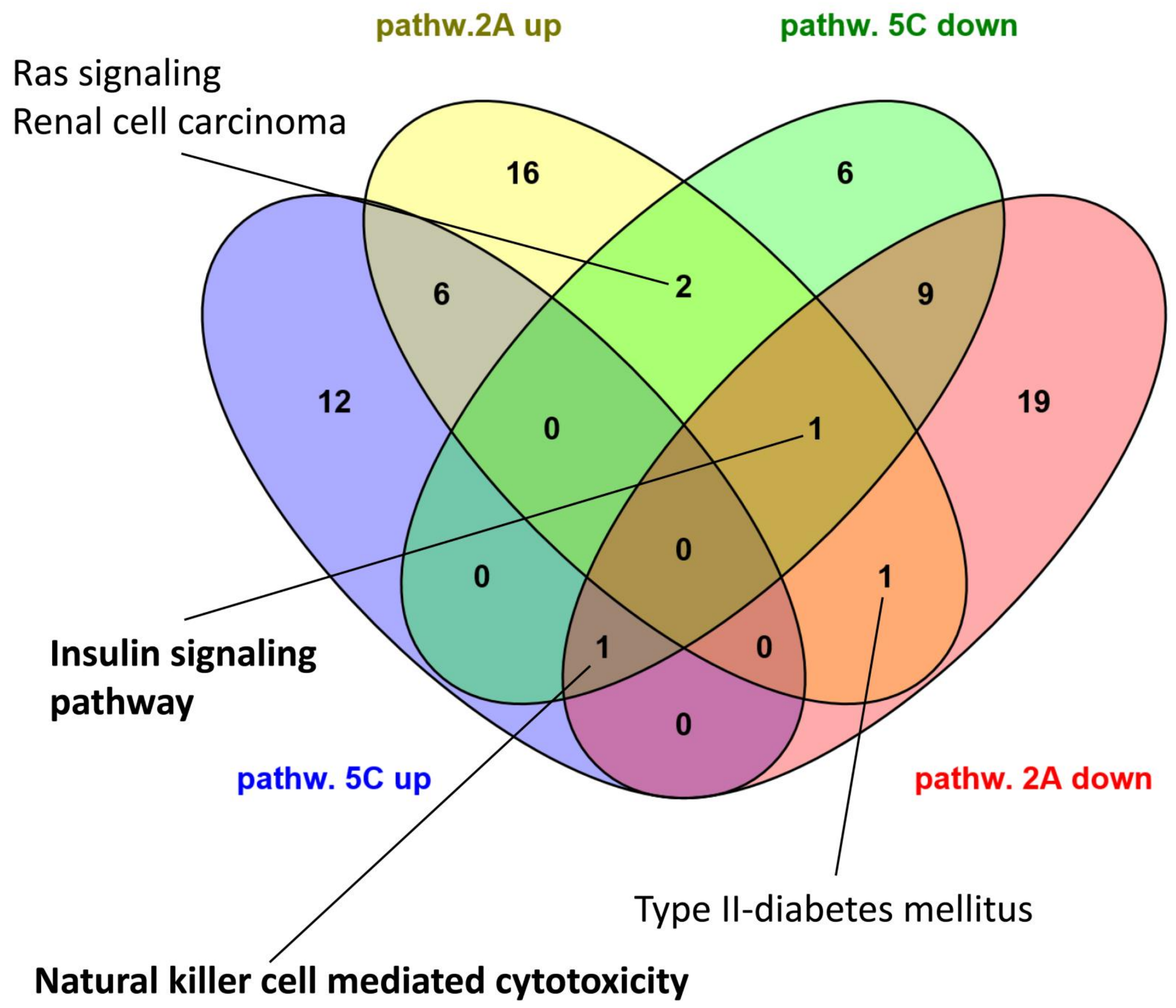

Supplementary Figure 5: KEGG pathways identified in miRNA functional enrichment analyses. **A.** 5C cell-specific enriched pathways stratified by up (red)- and down (green)-regulated miRNA subsets. The yellow node represents pathways enriched for both up- and down-regulated miRNA subsets. **B.** 2A cell-specific enriched pathways stratified by up (red)- and down (green)-regulated miRNA subsets. Yellow nodes represent pathways enriched for both up- and down-regulated miRNA subsets. The node size reflects the number of pathway-related genes regulated by the specific miRNA set. The edge weight illustrates the degree of gene overlap between two pathways as measured by the Jaccard index. Pathways highlighted in bold are enriched in both AI-resistant cell models. The font size reflects the unadjusted

Fisher test P-values from the enrichment analysis ($P < 0.001$, large (24 pts); $0.001 \leq P < 0.01$, intermediate (18 pts); $0.01 \leq P < 0.05$, small (12 pts)). **C.** Enriched pathways of 5C- and 2A-specific up- and down-regulated miRNA subsets. X-axes represent -$\log_{10}$ value, Y-axes: enriched pathways sorted according to specific pathways enriched in 5C or 2A. **D.** Venn diagram illustrating pathway overlaps between up- and down-regulated miRNA sets of 5C and 2A cells (Table S1 and S2).

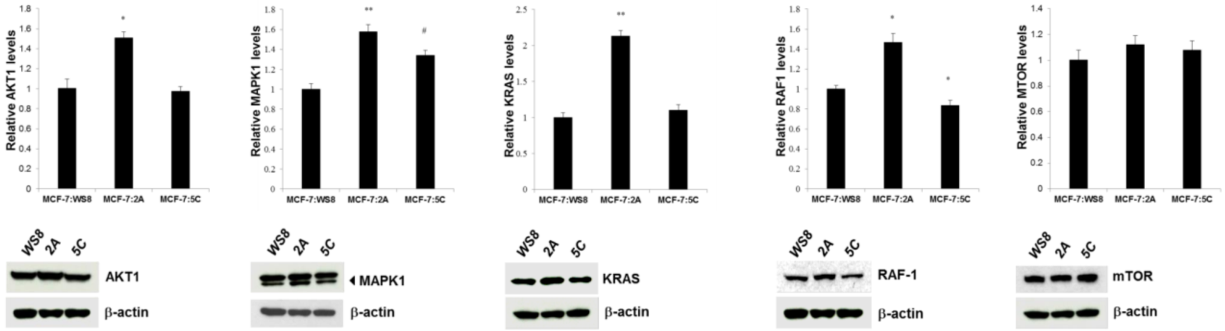

2A (FC)	5C (FC)
-	409-3p (76.90)
3185 (1/3.36)<br>-<br>374b (1/2.77)	3185 (1/4.68)<br>1285 (1/2.36)<br>-

2A (FC)	5C (FC)
-<br>-<br>-<br>-<br>-<br>-<br>-<br>-<br>1226 (2.47)<br>-<br>-<br>-<br>675 (3.97)<br>708 (3.22)<br>1273d (2.43)	409-3p (76.9)<br>487a (42.78)<br>433 (33.39)<br>31 (28.67)<br>409-5p (21.69)<br>543 (19.49)<br>370 (10.03)<br>494 (6.27)<br>1226 (3.28)<br>301a (2.71)<br>181d (2.54)<br>330-3p (2.54)<br>-<br>-<br>-
-<br>1246 (1/5.98)<br>3185 (1/3.36)<br>361-5p (1/2.35)<br>-<br>-<br>-	708 (1/10.79)<br>1246 (1/5.77)<br>3185 (1/4.68)<br>361-5p (1/2.28)<br>193a-5p (1/2.17)<br>210 (1/2.14)<br>7-1* (1/2.1)

2A (FC)	5C (FC)
-<br>-<br>-<br>-<br>-<br>-<br>671-5p (2.18)	409-3p (76.9)<br>543 (19.49)<br>329 (18.76)<br>493 (13.35)<br>376c (10.65)<br>181d (2.54)<br>-
1246 (1/5.98)<br>3185 (1/3.36)<br>27b (1/2.59)<br>181a-2* (1/7.64)<br>628-3p (1/2.24)<br>-<br>30e (1/2.54)<br>-<br>-<br>-<br>374b (1/2.77)<br>30a* (1/13.33)<br>30a (1/3.73)	1246 (1/5.77)<br>3185 (1/4.68)<br>27b (1/3.91)<br>181a-2* (1/3.62)<br>628-3p (1/2.36)<br>30b (1/2.24)<br>30e (1/2.13)<br>200b (1/2.12)<br>7-1* (1/2.1)<br>30d (1/2.02)<br>-<br>-<br>-

2A (FC)	5C (FC)
-<br>-<br>-<br>-<br>1226 (2.47)<br>503 (2.21)<br>-<br>1910 (2.11)	134 (24.79)<br>329 (18.76)<br>493 (13.35)<br>370 (10.03)<br>1226 (3.28)<br>503 (3.26)<br>330-3p (2.54)<br>-
125b (1/16.15)<br><br><br>3185 (1/3.36)	125b (1/35.27)<br>195 (1/2.09)<br>497 (1/2.65)<br>3185 (1/4.68)<br>328 (1/4.59)<br>1231 (1/4.07)<br>550 (1/2.13)

2A (FC)	5C (FC)
671-5p (2.18)<br>-	-<br>375 (3.33)
99a (1/11.3)<br>1246 (1/5.98)<br>-<br>30a (1/3.73)	99a (1/44.62)<br>1246 (1/5.77)<br>193a-5p (1/2.17)<br>-

Supplementary Figure 6: Basal levels of gene and protein expression of key growth factor signal transduction mediators compared between AI resistance models 2A and 5C and their modulatory miRNAs. Gene expression levels were quantified by RT-PCR and protein expression levels were examined by Western blotting with ß-action as loading control (WS8: E2 growth sensitive control). Up (red) and down (green) regulating miRNAs were obtained from specific miRNA sets given in Table 1. Putative miRNA-mRNA interaction thresholds were defined as CLIP confirmed Targetscan 7.0 in silico predictions (> 50 percentile). MiRNAs of the DLK-DIO3 cluster of chromosome 14 are underlined. Small down regulatory effects in 5C compared to 2A were observed for AKT1, MAPK1, KRAS, RAF-1. Small

up regulatory effects in 5C compared to 2A were observed for MTOR. *: $P < 0.05$, #: $P < 0.01$, **: $P < 0.001$ (comparisons to WS8).

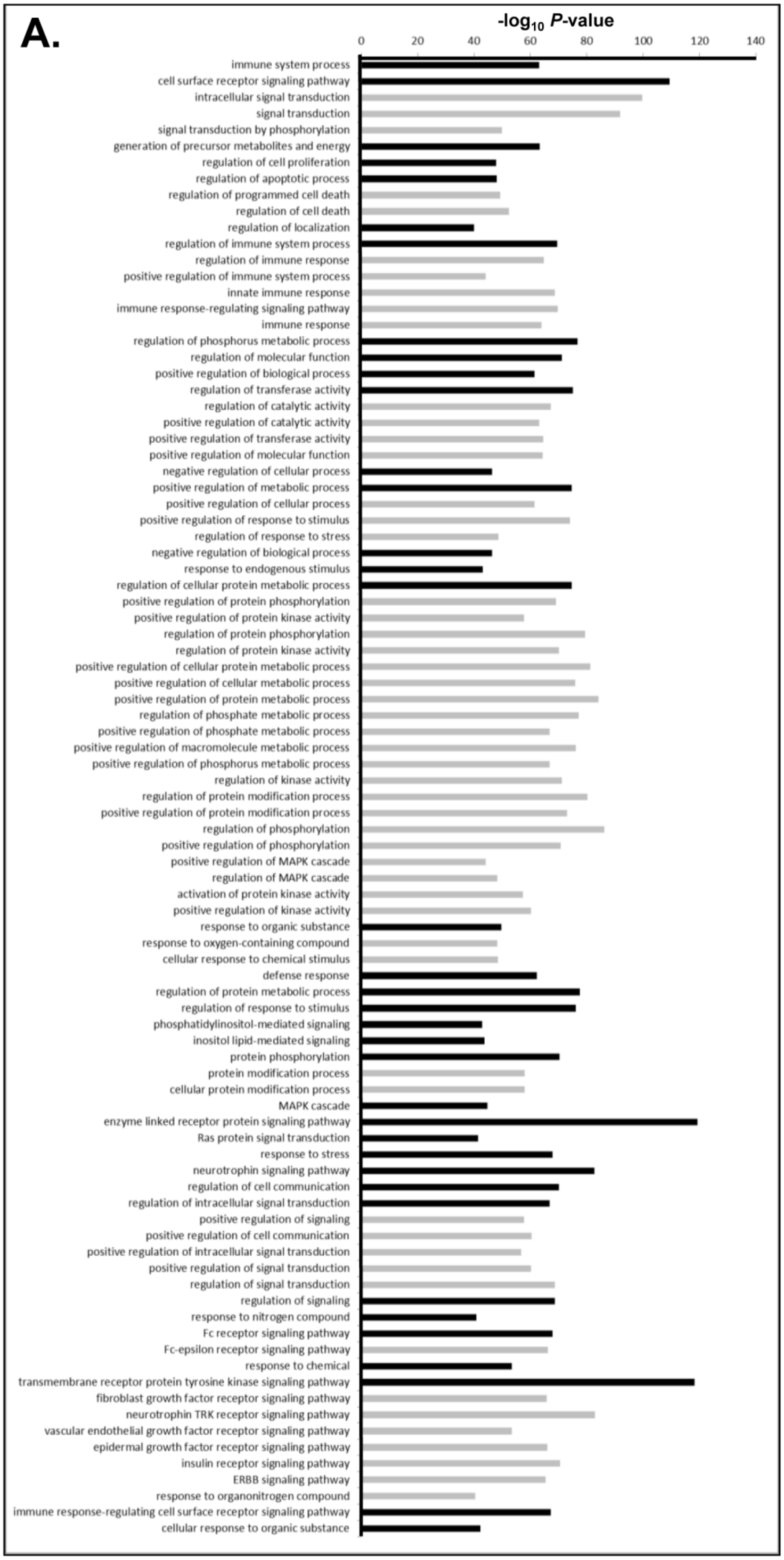
A.
-log10 P-value
0
20
40
60
80
100
120
140
immune system process
cell surface receptor signaling pathway
intracellular signal transduction
signal transduction
signal transduction by phosphorylation
generation of precursor metabolites and energy
regulation of cell proliferation
regulation of apoptotic process
regulation of programmed cell death
regulation of cell death
regulation of localization
regulation of immune system process
regulation of immune response
positive regulation of immune system process
innate immune response
immune response-regulating signaling pathway
immune response
regulation of phosphorus metabolic process
regulation of molecular function
positive regulation of biological process
regulation of transferase activity
regulation of catalytic activity
positive regulation of catalytic activity
positive regulation of transferase activity
positive regulation of molecular function
negative regulation of cellular process
positive regulation of metabolic process
positive regulation of cellular process
positive regulation of response to stimulus
regulation of response to stress
negative regulation of biological process
response to endogenous stimulus
regulation of cellular protein metabolic process
positive regulation of protein phosphorylation
positive regulation of protein kinase activity
regulation of protein phosphorylation
regulation of protein kinase activity
positive regulation of cellular protein metabolic process
positive regulation of cellular metabolic process
positive regulation of protein metabolic process
regulation of phosphate metabolic process
positive regulation of phosphate metabolic process
positive regulation of macromolecule metabolic process
positive regulation of phosphorus metabolic process
regulation of kinase activity
regulation of protein modification process
positive regulation of protein modification process
regulation of phosphorylation
positive regulation of phosphorylation
positive regulation of MAPK cascade
regulation of MAPK cascade
activation of protein kinase activity
positive regulation of kinase activity
response to organic substance
response to oxygen-containing compound
cellular response to chemical stimulus
defense response
regulation of protein metabolic process
regulation of response to stimulus
phosphatidylinositol-mediated signaling
inositol lipid-mediated signaling
protein phosphorylation
protein modification process
cellular protein modification process
MAPK cascade
enzyme linked receptor protein signaling pathway
Ras protein signal transduction
response to stress
neurotrophin signaling pathway
regulation of cell communication
regulation of intracellular signal transduction
positive regulation of signaling
positive regulation of cell communication
positive regulation of intracellular signal transduction
positive regulation of signal transduction
regulation of signal transduction
regulation of signaling
response to nitrogen compound
Fc receptor signaling pathway
Fc-epsilon receptor signaling pathway
response to chemical
transmembrane receptor protein tyrosine kinase signaling pathway
fibroblast growth factor receptor signaling pathway
neurotrophin TRK receptor signaling pathway
vascular endothelial growth factor receptor signaling pathway
epidermal growth factor receptor signaling pathway
insulin receptor signaling pathway
ERBB signaling pathway
response to organonitrogen compound
immune response-regulating cell surface receptor signaling pathway
cellular response to organic substance

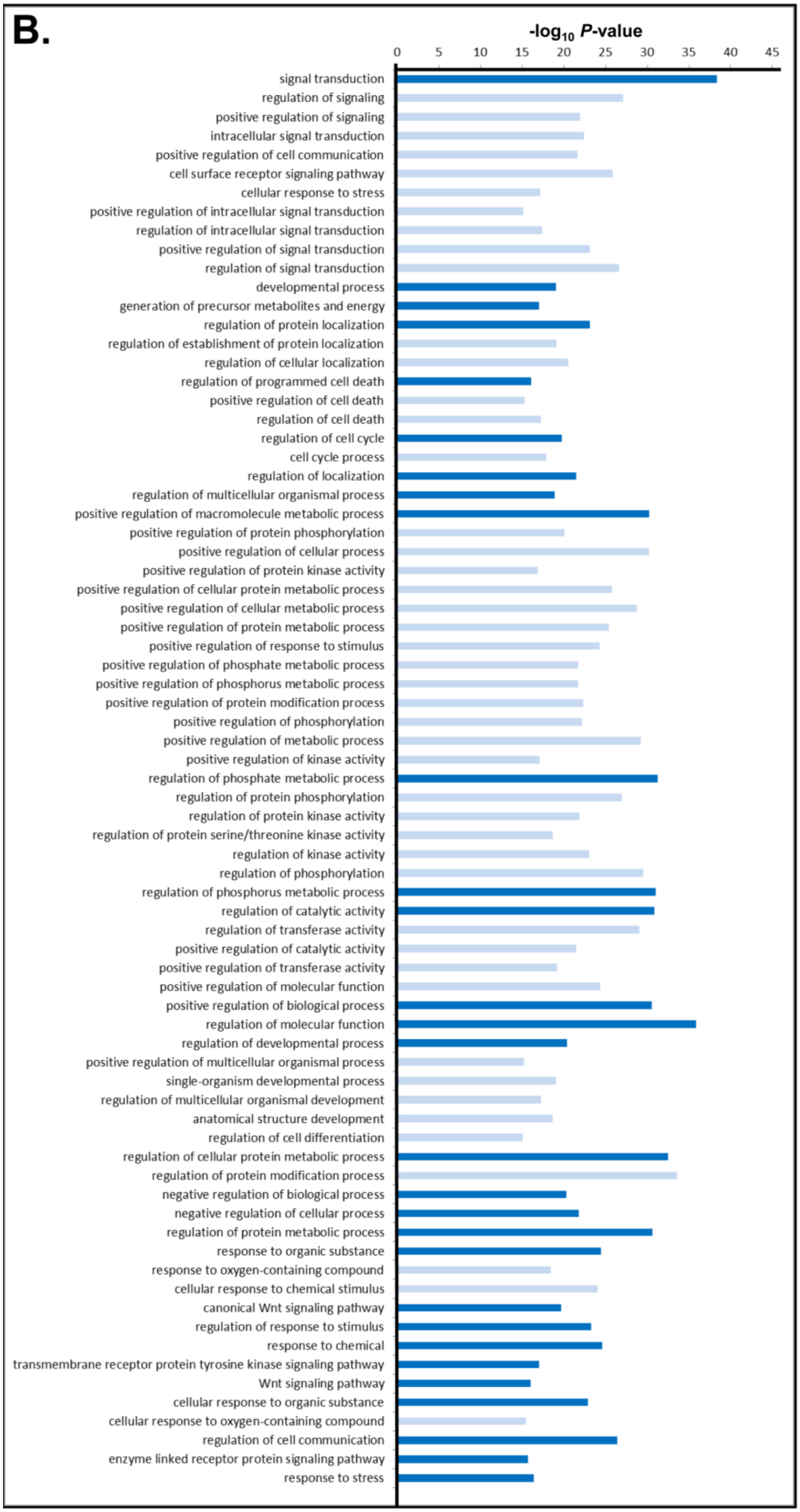
B.
-log10 P-value
0
5
10
15
20
25
30
35
40
45
signal transduction
regulation of signaling
positive regulation of signaling
intracellular signal transduction
positive regulation of cell communication
cell surface receptor signaling pathway
cellular response to stress
positive regulation of intracellular signal transduction
regulation of intracellular signal transduction
positive regulation of signal transduction
regulation of signal transduction
developmental process
generation of precursor metabolites and energy
regulation of protein localization
regulation of establishment of protein localization
regulation of cellular localization
regulation of programmed cell death
positive regulation of cell death
regulation of cell death
regulation of cell cycle
cell cycle process
regulation of localization
regulation of multicellular organismal process
positive regulation of macromolecule metabolic process
positive regulation of protein phosphorylation
positive regulation of cellular process
positive regulation of protein kinase activity
positive regulation of cellular protein metabolic process
positive regulation of cellular metabolic process
positive regulation of protein metabolic process
positive regulation of response to stimulus
positive regulation of phosphate metabolic process
positive regulation of phosphorus metabolic process
positive regulation of protein modification process
positive regulation of phosphorylation
positive regulation of metabolic process
positive regulation of kinase activity
regulation of phosphate metabolic process
regulation of protein phosphorylation
regulation of protein kinase activity
regulation of protein serine/threonine kinase activity
regulation of kinase activity
regulation of phosphorylation
regulation of phosphorus metabolic process
regulation of catalytic activity
regulation of transferase activity
positive regulation of catalytic activity
positive regulation of transferase activity
positive regulation of molecular function
positive regulation of biological process
regulation of molecular function
regulation of developmental process
positive regulation of multicellular organismal process
single-organism developmental process
regulation of multicellular organismal development
anatomical structure development
regulation of cell differentiation
regulation of cellular protein metabolic process
regulation of protein modification process
negative regulation of biological process
negative regulation of cellular process
regulation of protein metabolic process
response to organic substance
response to oxygen-containing compound
cellular response to chemical stimulus
canonical Wnt signaling pathway
regulation of response to stimulus
response to chemical
transmembrane receptor protein tyrosine kinase signaling pathway
Wnt signaling pathway
cellular response to organic substance
cellular response to oxygen-containing compound
regulation of cell communication
enzyme linked receptor protein signaling pathway
response to stress

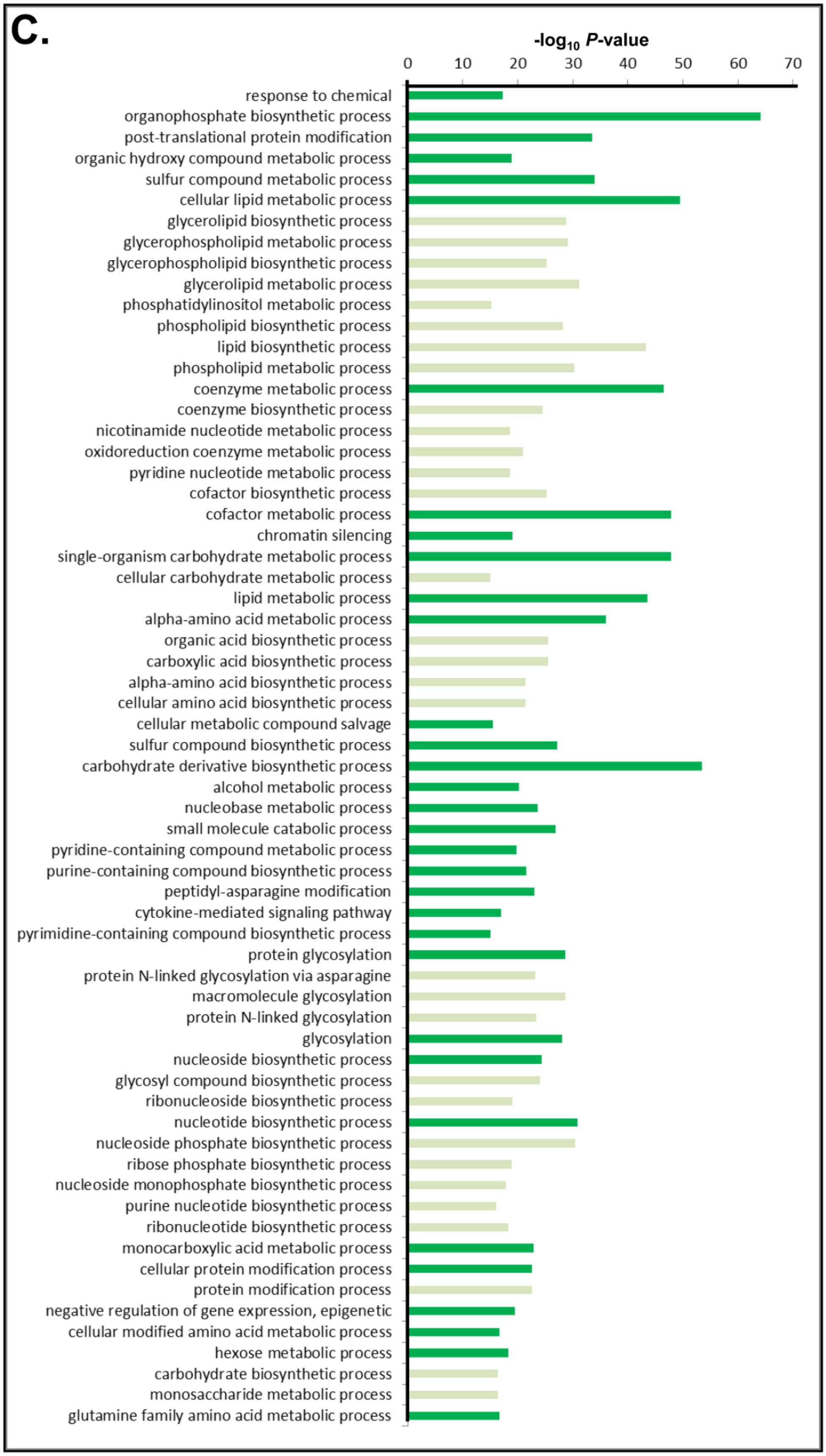
C.
-log10 P-value
0
10
20
30
40
50
60
70
response to chemical
organophosphate biosynthetic process
post-translational protein modification
organic hydroxy compound metabolic process
sulfur compound metabolic process
cellular lipid metabolic process
glycerolipid biosynthetic process
glycerophospholipid metabolic process
glycerophospholipid biosynthetic process
glycerolipid metabolic process
phosphatidylinositol metabolic process
phospholipid biosynthetic process
lipid biosynthetic process
phospholipid metabolic process
coenzyme metabolic process
coenzyme biosynthetic process
nicotinamide nucleotide metabolic process
oxidoreduction coenzyme metabolic process
pyridine nucleotide metabolic process
cofactor biosynthetic process
cofactor metabolic process
chromatin silencing
single-organism carbohydrate metabolic process
cellular carbohydrate metabolic process
lipid metabolic process
alpha-amino acid metabolic process
organic acid biosynthetic process
carboxylic acid biosynthetic process
alpha-amino acid biosynthetic process
cellular amino acid biosynthetic process
cellular metabolic compound salvage
sulfur compound biosynthetic process
carbohydrate derivative biosynthetic process
alcohol metabolic process
nucleobase metabolic process
small molecule catabolic process
pyridine-containing compound metabolic process
purine-containing compound biosynthetic process
peptidyl-asparagine modification
cytokine-mediated signaling pathway
pyrimidine-containing compound biosynthetic process
protein glycosylation
protein N-linked glycosylation via asparagine
macromolecule glycosylation
protein N-linked glycosylation
glycosylation
nucleoside biosynthetic process
glycosyl compound biosynthetic process
ribonucleoside biosynthetic process
nucleotide biosynthetic process
nucleoside phosphate biosynthetic process
ribose phosphate biosynthetic process
nucleoside monophosphate biosynthetic process
purine nucleotide biosynthetic process
ribonucleotide biosynthetic process
monocarboxylic acid metabolic process
cellular protein modification process
protein modification process
negative regulation of gene expression, epigenetic
cellular modified amino acid metabolic process
hexose metabolic process
carbohydrate biosynthetic process
monosaccharide metabolic process
glutamine family amino acid metabolic process

Supplementary Figure 7: GO terms of the subcategory 'Biological Process' identified in Gene Set Enrichment Analyses with the GORILLA tool. Test set gene lists were obtained from KEGG pathway interaction networks (Figs. 5A, B). Resulting GO terms were summarized by removing redundant terms with REVIGO using default parameters. Terms were then filtered according to the following thresholds: frequency < 5% and $-\log_{10} P < 40$ (intersection gene set), $-\log_{10} P < 15$ (5C-, 2A-specific gene sets). Dark color bars represent major GO terms; light color bars specify minor sub-terms of major terms (bold). **A.** Intersection of 5C versus 2A gene sets. **B.** 5C-specific gene set. **C.** 2A-specific gene set. X-axes: adjusted $-\log_{10}$ *P*-Value; Y-axes: GO terms.

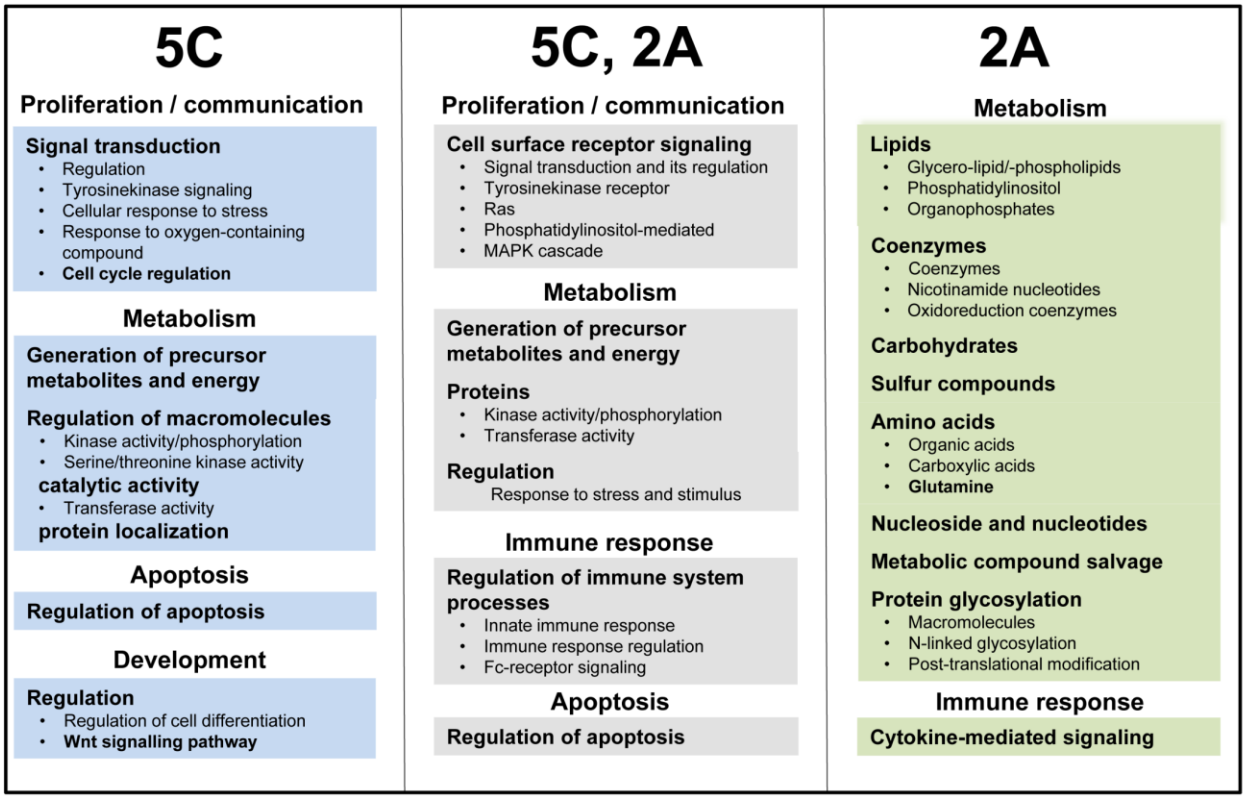

Supplementary Figure 8: Abbreviated GO terms of the subcategory 'Biological process' enriched in 5C and 2A models. Major (bold) and minor terms are listed for the AI resistance phenotype common to the 5C and 2A models (grey boxes), 5C-specific (blue boxes) and 2A-specific phenotype (green boxes).

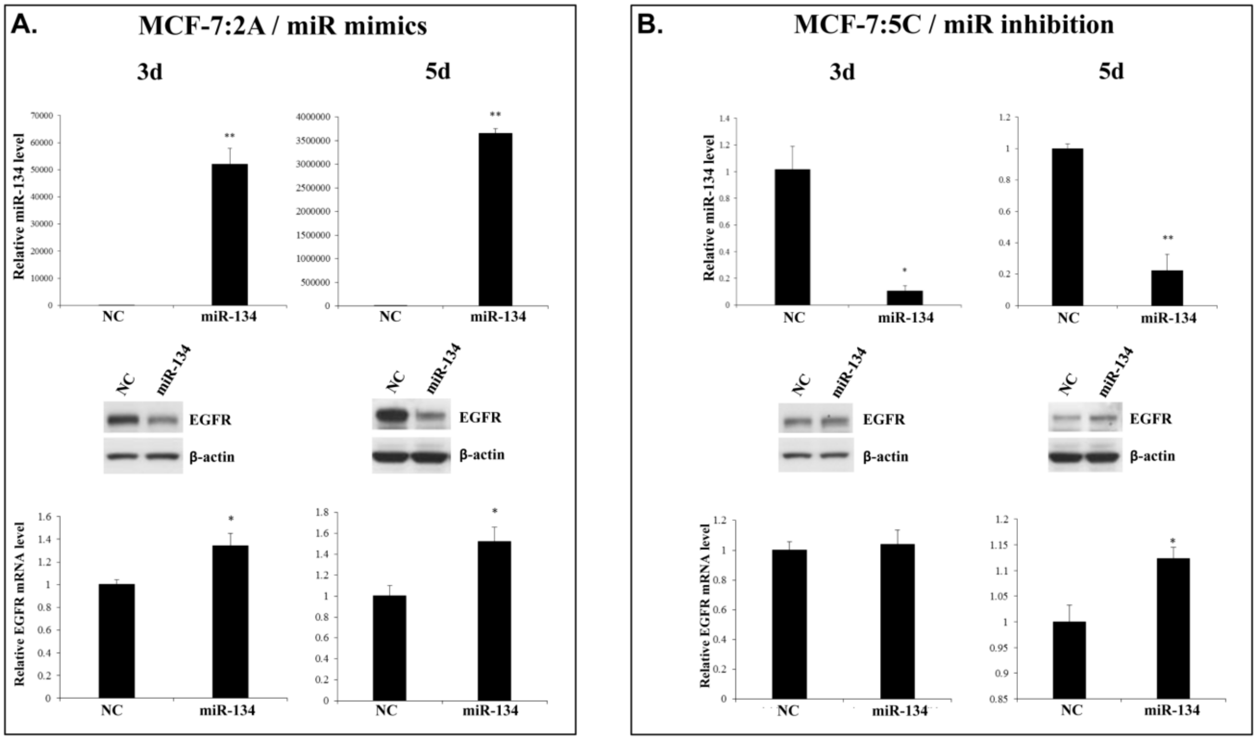

Supplementary Figure 9: miR-134 mimic and inhibition affect EGFR expression in MCF-7:2A and MCF-7:5C cells, respectively. A. In 2A cells miR-134 expression levels are significantly increased at 3 and 5 days. Although EGFR mRNA levels are increased, the downregulation of EGFR protein correlates with relative miR-134 levels. **B.** In 5C cells miR-134 expression levels are significantly decreased at 3 and 5 day inhibition. EGFR mRNA level is significantly increased after 5 days which correlates with an increase of EGFR protein. ß-actin was used as loading control; NC: negative control. qRT-PCR experiments were done in triplicates; *: $P < 0.05$, **: $P < 0.001$.
